# RSK2-mediated cGAS phosphorylation induces cGAS chromatin-incorporation-mediated cell transformation and cancer cell colony growth

**DOI:** 10.1038/s41420-024-02208-8

**Published:** 2024-10-18

**Authors:** Weidong Chen, Ga-Eun Lee, Dohyun Jeung, Jiin Byun, Juan Wu, Xianzhe Li, Joo Young Lee, Han Chang Kang, Hye Suk Lee, Kwang Dong Kim, Soo-Bin Nam, Cheol-Jung Lee, Young Jik Kwon, Yong-Yeon Cho

**Affiliations:** 1 BK21-Four, College of Pharmacy, The Catholic University of Korea, 43, Jibong-ro, Wonmi-gu, Bucheon-si, Gyeonggi-do 14662 Korea; 2 Biopharmaceutical Research Center, Ochang Institute of Biological and Environmental Sciences, Korea Basic Science Institute, 162, Yeongudanji-ro, Ochang-eup, Cheongwon-gu, Cheongju-si 28119 Republic of Korea; 3 Research Institute for Controls and Materials of Regulated Cell Death, The Catholic University of Korea, 43, Jibong-ro, Wonmi-gu, Bucheon-si, Gyeonggi-do 14662 Korea; 4 College of Pharmacy, The Catholic University of Korea, 43, Jibong-ro, Wonmi-gu, Bucheon-si, Gyeonggi-do 14662 Korea; 5 Division of Applied Life Science (BK21 four), PMBBRC, Gyeongsang National University, Jinju, 52828 Korea; 6 Department of Pharmaceutical Sciences, University of California, 132, Sprague Hall, Irvine, CA 92697 USA

**Keywords:** Chromatin remodelling, Kinases

## Abstract

Cyclic guanosine-adenosine monophosphate synthase (cGAS) is a key cytosolic DNA sensor that plays a pivotal role in the innate immune response. Although a decade of research on the cGAS has advanced our understanding of inflammasome formation, cytokine production, and signaling pathways, the role of cGAS in the nucleus remains unclear. In this study, we found that the nuclear localization of endogenous and stably expressed cGAS differed from transiently expressed cGAS, which mainly localized in the cytosol. In the nucleus, cGAS is tightly bound to chromatin DNA. The chromatin DNA binding of cGAS was dependent on RSK2. Our molecular mechanism study indicated that the N-lobe of RSK2 harboring 1–323 interacted with the NTase domain of cGAS harboring residues 213–330. This interaction increased RSK2-induced cGAS phosphorylation at Ser120 and Thr130, resulting in the tightly binding of cGAS to chromatin. Importantly, epidermal growth factor (EGF)-induced cell transformation and anchorage-independent colony growth showed an increase in growth factors, such as EGF or bFGF, in cGAS stable expression compared to mock expression. Notably, the cGAS-S120A/T130A mutant abolished the increasing effect of cell transformation of JB6 Cl41 cells and colony growth of SK-MEL-2 malignant melanoma cells. The results suggested that cGAS’s chromatin DNA binding, which is indispensable to RSK2-dependent phosphorylation of cGAS at Ser120/Thr130, provides the first clue to how cGAS may participate in chromatin remodeling in the nucleus.

## Introduction

Cyclic guanosine monophosphate-adenosine monophosphate synthase (cGAS), an enzyme catalyzing the reaction that syntheses the production of cyclic guanosine monophosphate (GMP)-adenosine monophosphate (AMP) (cGAMP), is canonically known to predominantly localize in the cytoplasm, where it binds to double-stranded DNA (dsDNA) in a sequence-independent [1] and a length-dependent manner [2]. cGAS plays a pivotal role in the innate immune response by detecting foreign DNA from infected viruses/bacteria [3–5] and damaged or aberrant self-DNA from the nucleus and mitochondria [6–8]. Due to these reasons, the role and signaling pathway for cGAS have primarily focused on activating the stimulator of interferon genes (STING), leading to the production of type I interferons and inflammatory cytokines [1, 6, 7, 9]. When cGAS was first identified as a cytosolic DNA sensor in 2006, the key challenge was understanding how its sequence-independent sensing, triggered by the DNA sugar-phosphate backbone, could avoid self-reactivity against genomic DNA in nucleated cells. Following this discovery, research on cytosolic cGAS accelerated, particularly as cGAS and STING were identified as crucial adaptors in NF-κB-mediated inflammatory responses. Notably, most research published prior to 2019 focused on the cytosolic role of cGAS, often using transient expression methodologies. However, after 2019, research began to explore the nuclear role of cGAS, as it was found that endogenous and stably expressed cGAS are primarily localized in the nucleus [10]. Moreover, cGAS has been shown to participate in DNA tethering at the nuclear membrane [10, 11], which aligns with the concepts in our study. Additionally, stimuli such as cisplatin and UVB, which directly damage genomic DNA, can lead to nuclear membrane rupture and genomic DNA leakage. In such situations, we hypothesize that cytosolic cGAS re-translocates into the nucleus, while residual cGAS in the cytoplasm continues to enhance immune responses through the cGAMP-STING pathway. This hypothesis was supported by which cGAS binding to chromatin was recently identified based on its DNA-binding properties [12], suggesting that cGAS may be redistributed to the cytosol during the resolution of cell division and the reformation of the nuclear envelope [12]. However, by now, is not clear the role of cGAS in the nucleus.

It has been known that histone proteins, especially H2A and H2B, play a key role in cGAS’s chromatin tethering. Histone proteins, which are rich in basic amino acids, interact strongly with negatively charged DNA to form nucleosomes. Similarly, cGAS contains basic amino acids that mediate DNA interaction. Notably, the positively charged amino acids Arg222, Lys240, and Arg241 in cGAS interact with the negatively charged residues Glu61, Asp90, and Glu92 of histone 2A [13]. This interaction results in cGAS binding to the nucleosome core particle and inhibiting its activity [13]. For cGAS to enzymatically synthesize cGAMP, a functionally active 2:2 cGAS-DNA oligomerization is required, involving two distinct DNA-binding sites, referred to as sites A and B [13]. However, the H2A-H2B dimer masks site B when cGAS interacts with the nucleosome, sterically preventing the formation of the active 2:2 cGAS-DNA complex, leading cGAS to adopt an inactive conformation [13]. Besides structural interference for the cGAS activity regulation by nucleosome complex formation, phosphorylation of cGAS at Tyr215 by B-lymphoid tyrosine kinase promotes nuclear accumulation of cGAS when cells are exposed to etoposide [14]. Interestingly, cGAS knockdown in non-small cell lung carcinoma suppresses tumor growth in a xenograft mouse model, indicating that cGAS promotes tumorigenesis [14]. However, no molecular mechanisms of how cGAS participates in tumorigenesis have been elucidated yet.

RSK2, a member of the ribosomal S6 kinase (RSK) family of enzymes, plays critical roles in cellular processes, including cell growth, cell mortality, cell survival, and cell proliferation [15]. RSK2 is activated by an extracellular signal-regulated kinase (ERK) in response to various extrinsic stimuli, including growth factors, hormones, neurotransmitters, and ultraviolets (UV) [15]. Since RSK2 has been known to phosphorylate a wide range of substrates that regulate apoptosis, cell cycle regulation, and gene expression involved in cell survival and proliferation, it has become an emerging target for therapeutic intervention [15, 16]. For example, RSK2 deficiency abolished epidermal growth factor (EGF)-induced histone H3 phosphorylation, and re-introduction of RSK2 into RSK2 deficient Coffin-Lowry Syndrome patient cells restores EGF-induced histone H3 phosphorylation at Ser10 [17], indicating that RSK2 acts as a kinase to induce chromatin remodeling in gene expression process. Our previous studies indicated that RSK2 directly phosphorylate histone 2B at Ser32, resulting in the increase of cell transformation induced by EGF [18]. Moreover, H2AX phosphorylation at Ser139 mediated by RSK2 and DNA-PK, but not ATM or ATR, suppressed RSK2-mediated histone H3 at Ser10 [19]. Interestingly, RSK2-mediated H2AX phosphorylation at Ser139 and Ser16 prevents H2AX degradation when the cells are irradiated by ultraviolet B (UVB). Importantly, EGF-associated AP-1 transactivation activity was dramatically lower in H2AX^−/−^/H2AX-wt cells than H2AX^−/−^/H2AX-AA, indicating that RSK2/H2AX signaling pathway negatively regulates the RSK2/histone H3 pathway and thus maintains normal cell proliferation [19]. Since recent results demonstrated that wiring AKT-RSK2 signaling regulates H2AX Ser16 phosphorylation [20], resulting in increase of oncogenic activity by inducing G_1_/S cell cycle transition [21]. These results strongly suggested that RSK2 plays an essential role in chromatin remodeling. However, the mechanism for the RSK2-mediated chromatin remodeling has been barely elucidated.

## Results

### cGAS is a binding partner of RSK2 in the nucleus

**Fig. 1:**
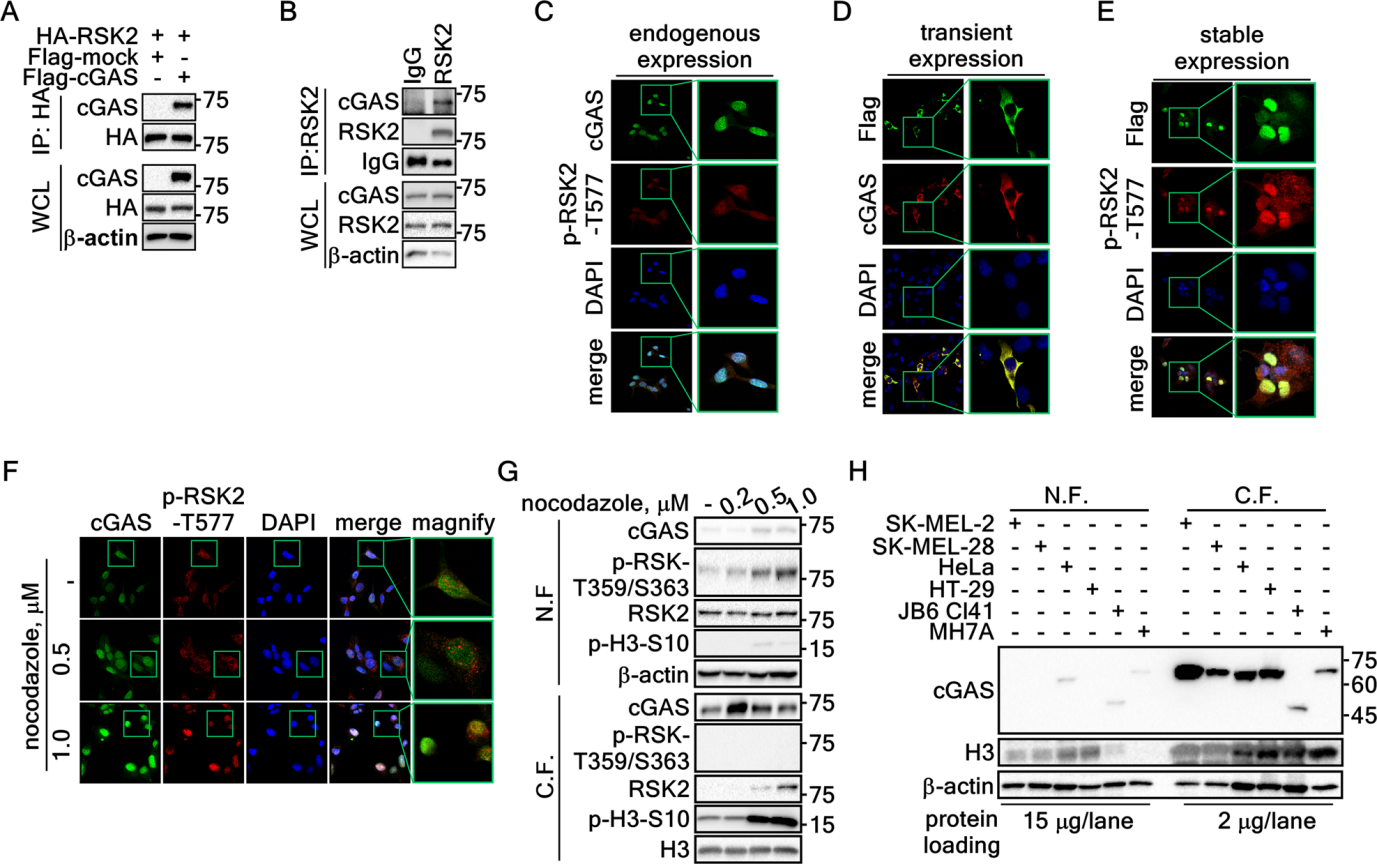
RSK2 and cGAS colocalization and interaction in the nucleus. **A** IP/Western blots illustrating the interaction between cGAS and RSK2 in SK-MEL-2 cells stably expressing HA-RSK2 and Flag-cGAS as indicated. WCL, whole cell lysate. **B** IP and Western blots illustrating the endogenous interaction between cGAS and RSK2 in SK-MEL-2 cells. **C** Immunocytofluorescence (ICF) images showing colocalization of endogenously expressed cGAS and RSK2 in the nucleus. SK-MEL-2 cells were stained with specific antibodies against cGAS and active RSK2. Confocal microscopy (×200) detected by Alexa Fluor 488 for cGAS, Alexa Fluor 568 for p-RSK2-T577, DAPI for nuclei. Boxed area, magnified. **D** ICF images showing colocalization of transiently overexpressed cGAS in the cytosol. Confocal microscopy (×200) detected by Alexa Fluor 488 for Flag, Alexa Fluor 568 for cGAS, DAPI for nuclei. Boxed area, magnified. **E** ICF images showing colocalization of stably overexpressed cGAS and active RSK2 in the nucleus. Confocal microscopy (×200) detected by Alexa Fluor 488 for Flag-cGAS, Alexa Fluor 568 for p-RSK2-T577, DAPI for nuclei. Boxed area, magnified. **F** ICF images illustrating the increase of endogenous cGAS and active RSK2 accumulation by nocodazole in a dose-dependent manner. Confocal microscopy (×200) detected by Alexa Fluor 488 for cGAS, Alexa Fluor 568 for p-RSK2-T577, DAPI for nuclei. Boxed area, magnified. **G** Western blots illustrating that nocodazole increased cGAS and RSK2 protein in chromatin-bound fraction. **H** Western blots illustrating that cGAS expression is observed in the nuclear fraction of normal and cancer cell lines. **G,H** RIPA cell lysis buffer (1% Triton X-100, 0.1% SDS, 0.5% sodium deoxycholate, 50 mM Tris-HCl (pH 7.4), 150 mM NaCl, and 2 mM EDTA) was used to divide the protein lysates as non-chromatin-bound fraction (N.F.) and chromatin-bound fraction (C.F.). β-actin and H3 were used as internal controls for non-chromatin bound fraction and chromatin-bound fraction, respectively.

Our previous results demonstrated that RSK2 is involved in diverse cell processes, including carcinogenesis, inflammation and immune responses, and chromatin remodeling [21–23]. At the initiation of this study for the identification of potential RSK2 substrates in nuclear membrane integrity-mediated chromatin remodelling and DAMP-mediated immune and inflammatory reactions, cGAS caught our pay attention. In initiation, we analyzed the amino acid sequence of cGAS to identify the consensus target motifs, RxRxxS/T or RxxS/T, for RSK2-mediated phosphorylation [24, 25]. The result showed that cGAS contained potential 5 RSK2-mediated phosphorylation motifs (Supplementary Fig. 1A). This information motivated us to confirm the interaction between RSK2 and cGAS. The interaction between cGAS and RSK2 was confirmed by IP using cell lysates expressing Flag-cGAS and HA-RSK2 (Fig. 1A, Supplementary Fig. 1B). Moreover, the interaction of endogenous cGAS and RSK2 was also confirmed (Fig. 1B), indicating that cGAS and RSK2 interaction occurs in physiological condition. To confirm the subcellular location where RSK2 and cGAS interact, we examined the co-localization of RSK2 and cGAS by ICF assay. As expected, phosphorylated RSK2 at Thr577 was mainly detected in the nucleus (Fig. 1C) similar to our previous report [22]. Surprisingly, we observed that endogenous cGAS was mainly detected in the nucleus (Fig. 1C, Supplementary Fig. 1C) in contrast our expectation. Interestingly, while transiently expressed cGAS was mainly detected in the cytoplasm as expectation (Fig. 1D, Supplementary Fig. 1D, upper panels), stably expressed cGAS mainly showed the accumulation in the nucleus, similar to endogenous cGAS (Fig. 1E, Supplementary Fig. 1D, bottom panels). Additionally, the cytosolic accumulation of transiently expressed cGAS was observed not only in SK-MEL-2 cells but also in HEK293T cells (Supplementary Fig. 1D, upper panels). Previous results indicated that RSK2-mediated phosphorylation of H3 at Ser10 and H2B at Ser32 might involve in chromosome condensation in G_2_/M cell cycle phase [18, 26], where cGAS is tethered to chromatin and inactivated [27]. Thus, RSK2 may participate cGAS interaction in metaphase of cell cycle. The nocodazole-treated G_2_/M accumulation (Supplementary Fig. 1E, F) increased cGAS nuclear accumulation (Fig. 1F). Concurrently, p-RSK2-T577 levels also were increased in the nucleus by nocodazole treatment (Fig. 1F). Similarly, cGAS and RSK2 protein levels in chromatin fraction were dramatically increased by nocodazole treatment, while cGAS and RSK2 protein levels in non-chromatin fraction were not significantly changed (Fig. 1G). Interestingly, H3 Ser10 phosphorylation was increased by nocodazole, similar to RSK2 protein levels in the nucleus, while phosphorylation of RSK1 at Thr359/Ser363 was dramatically increased in the cytoplasm (Fig. 1G). We further confirmed that endogenous cGAS protein is highly detected in the nucleus of different cells, not only in cancer cells, such as SK-MEL-2, SK-MEL-28, HeLa, and HT-29, but also in not transformed cells, such as JB6 Cl41 and MH7A (Fig. 1H), indicating that cGAS protein is not mainly restricted in immune cells, but in every cells. Taken together, these results indicated that the interaction between cGAS and RSK2 in the nucleus is physiological phenomenon.

### Identification of interaction domains between cGAS and RSK2

**Fig. 2:**
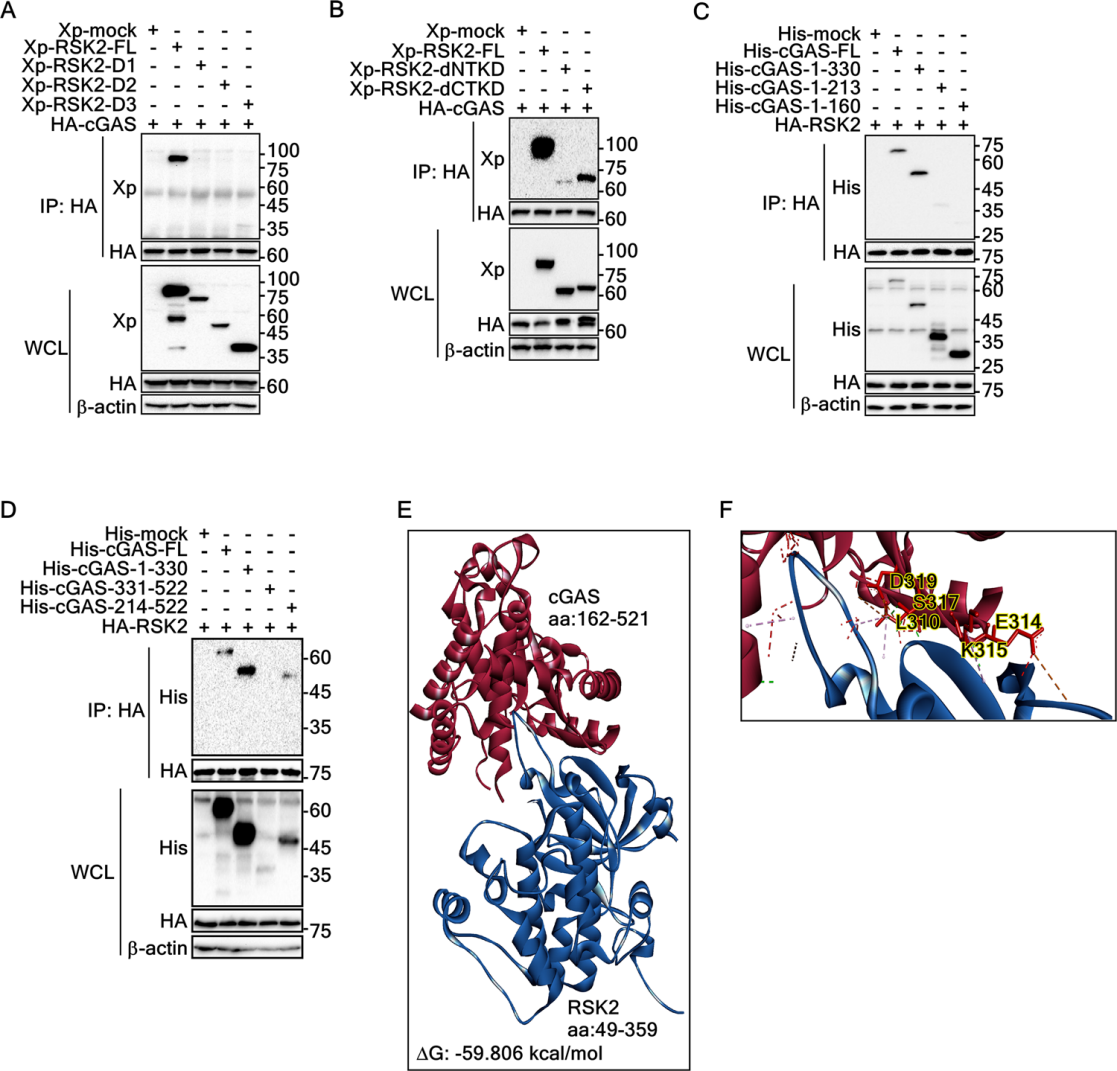
Identification of the interaction domains between RSK2 and cGAS. **A** IP/Western blot illustrating the determination of RSK2 domains interacting with cGAS in HEK293T cells transiently expressing Xp-RSK2 constructs and HA-cGAS as indicated. WCL, whole cell lysate. **B** IP/Western blots illustrating the determination of RSK2 kinase domain interacting with cGAS in HEK293T cells transiently expressing Xp-RSK2 constructs and HA-cGAS as indicated. WCL, whole cell lysate. **C** IP/Western blots illustrating the determination of cGAS domains interacting with RSK2 in HEK293T cells transiently expressing His-cGAS constructs and HA-RSK2 as indicated. WCL, whole cell lysate. **D** IP/Western blots illustrating the determination of cGAS domains interacting with RSK2 in HEK293T cells transiently expressing His-cGAS constructs and HA-RSK2 as indicated. WCL, whole cell lysate. **E** Illustration showing the overall protein-protein interaction between the NTKD of NTKD and amino acid 162-521 of cGAS using Discovery Studio (ver. 2021). The binding energy (∆*G*) is denoted as −59.806 kcal/mol. **F** Illustration showing the closed view of the interaction surface between RSK2 and cGAS for highlighting key amino acids involved in the interaction. Key amino acids of cGAS interacting with RSK2 are denoted. Detailed interaction information, including hydrogen bonds and hydrophobic and electrostatic interactions, is provided in Supplementary Table 1.

To identify the interaction domain of RSK2 with cGAS, we used RSK2 serial deletion mutants, designated as His-Xp-RSK2-FL, -D1, -D2, and -D3 (Supplementary Fig. 2A), which were cloned into a pcDNA4-His-Max expression vector [25]. IP using the cell lysate transiently expressing HA-cGAS and each of His-Xp-RSK2 deletion mutants (Supplementary Fig. 2A) showed a strong band in RSK2-FL, but not in any of deletion mutants (Fig. 2A), indicating that the N-terminal 1–68 amino acid of RSK2 might play a key role in cGAS binding. To confirm which kinase domain is involved in the interaction with cGAS, kinase domain deleted mutants of RSK2, His-Xp-RSK2-dNTKD and -dCTKD (Supplementary Fig. 2B), were utilized for IP. The strong RSK2 band in His-Xp-RSK2-FL disappeared in His-Xp-RSK2-dNTKD, not in His-Xp-RSK2-dCTKD (Fig. 2B), indicating that the NTKD of RSK2 plays a key role in the interaction of cGAS. In the opposite direction of the RSK2 and cGAS interaction, we constructed serial deletion constructs of cGAS from the C-terminal end, designated as His-Xp-cGAS-FL, -1–330. -1–213, and -1–160 (Supplementary Fig. 2C). The IP of cell lysates transiently expressing HA-RSK2 and each of cGAS deletion constructs showed that the bands observed in His-cGAS-FL and -1–330 disappeared in His-cGAS-1-213 (Fig. 2C), indicating the overlapping region of NTase and Mab21 domains spanning amino acids 213–330 plays an essential role in the interaction with RSK2-1–323. By IP with RSK2 and each of His-Xp-cGAS-FL, -1–330, -330–522, and -13–522 (Supplementary Fig. 2D), the bands observed in the His-Xp-cGAS-FL, -1–330, and -213–522 were not observed only in His-Xp-cGAS-330–522 (Fig. 2D), indicating that amino acids spanning 213–330 of cGAS play a key role in binding with RSK2. Based on these results, we proposed a model for the RSK2 and cGAS interaction. Protein-protein docking using Discovery Studio predicted seven hydrogen bonds five electrostatic interactions, and 4 hydrophobic interactions for the interaction between the NTKD of RSK2 and the 213–330 region of cGAS, with a binding score of −59.806 kcal/mol (Fig. 2E, Supplementary Table 1). The stability of the RSK2-cGAS complex, assessed through molecular dynamics simulations, indicated that the interaction between RSK2 and cGAS gradually approached a state of equilibrium and stability (Supplementary Fig. 2E). Notably, the total energy remained relatively constant through the simulation (Supplementary Fig. 2F), and similar fluctuation patterns measured by root-mean-square fluctuation (RMSF) (Supplementary Fig. 2G) indicated that the RSK2-cGAS complex exhibits stable binding by hydrogen bonds (L310, S317, and D319 of cGAS), electrostatic interaction (E314), and hydrophobic interaction (L315) (Fig. 2F). These results strongly indicate that the NTKD of RSK2 and the 213–330 region of cGAS play an essential role in the interaction between RSK2 and cGAS.

### cGAS incorporation into chromatin is mediated by RSK2

**Fig. 3:**
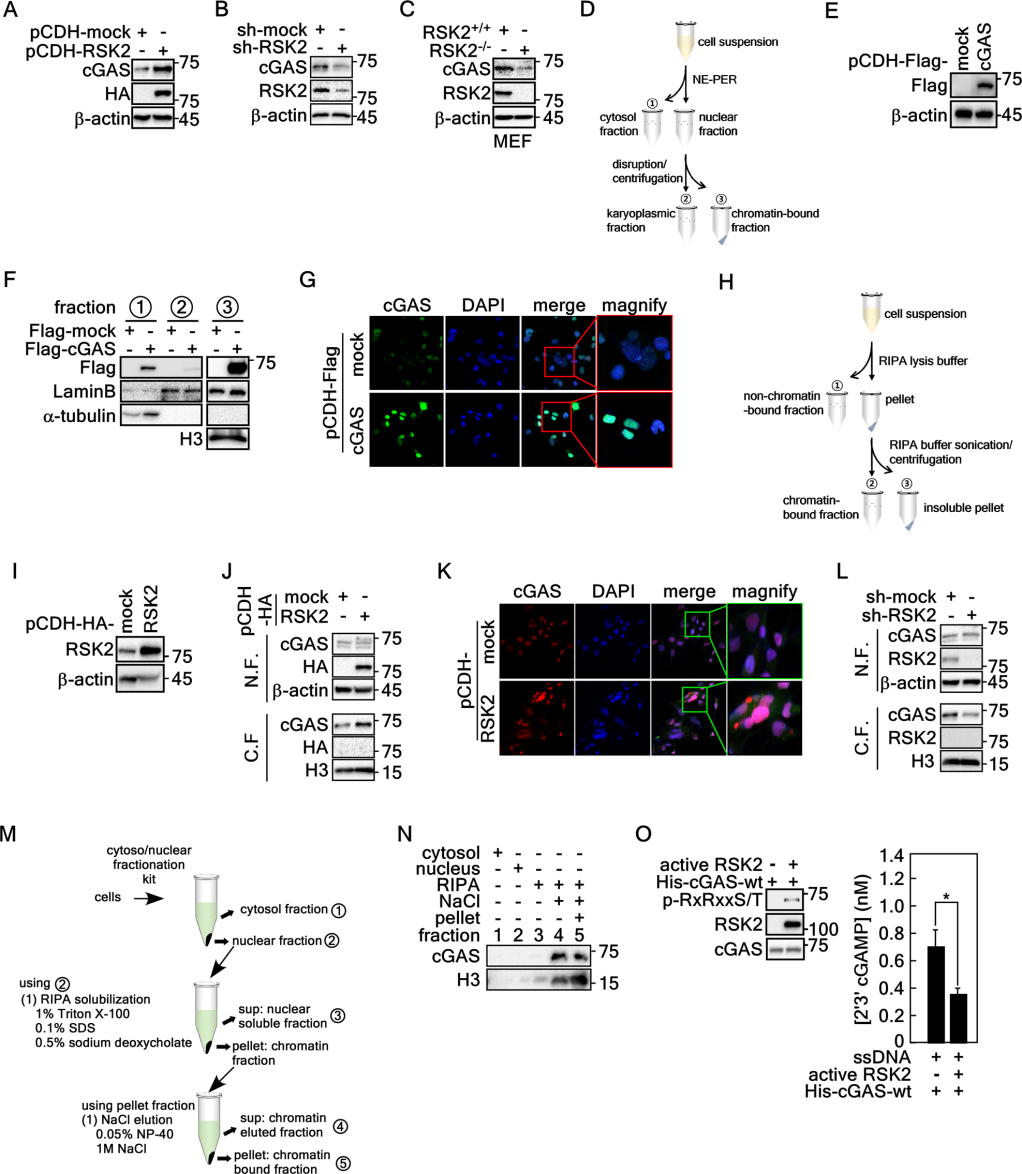
RSK2 increases cGAS chromatin incorporation. **A** Western blot illustrating that the stable expression of HA-RSK2 increases cGAS protein levels in SK-MEL-2 cells. **B** Western blots illustrate that RSK2 knockdown suppresses cGAS protein levels in SK-MEL-2 cells. **C** Western blots illustrate that RSK2 knockout mouse embryonic fibroblasts reduce cGAS protein levels. **D** Illustration depicting the protein extraction strategy for the cytosolic (fraction 1), karyoplasmic (fraction 2), and nuclear chromatin-bound (fraction 3) fractions by modifying the NE-PER nuclear and cytoplasmic extraction reagent kit. **E** Western blots confirming the stable expression of Flag-cGAS in SK-MEL-2 cells. **F** Western blots illustrate that Flag-cGAS protein is mainly detected in chromatin-bound fraction. The numbers in circles indicate the cytosolic fraction, karyoplasmic fraction, and chromatin-bound fraction, respectively. **G** ICF assay shows that stably expressing Flag-cGAS proteins are mainly localized in the nucleus. **H** Illustrations depicting the established simple methodology to separate the non-chromatin-bound fraction and chromatin-bound fraction. Cell lysate preparation by adding the RIPA buffer (1% Triton X-100, 0.1% SDS, 0.5% sodium deoxycholate, 50 mM Tris-HCl pH 7.4, 150 mM NaCl, and 2 mM EDTA) and centrifugation can separate non-chromatin-bound (fraction 1) and chromatin-bound fraction. The chromatin-bound fraction was used to prepare the chromatin-bound lysate (fraction 2) by RIPA re-solubilization and sonication. **I** Western blots confirm the stable overexpression of RSK2 in SK-MEL-2 cells. **J** Western blots illustrate that RSK2 overexpression increases cGAS protein levels in chromatin-bound fraction. N.F., non-chromatin-bound fraction; and C.F., chromatin-bound fraction. **K** ICF analysis illustrates that HA-RSK2 overexpression increases endogenous cGAS protein levels at the nucleus in SK-MEL-2 cells by confocal microscopy. **L** Western blots illustrate that RSK2 knockdown suppresses endogenous cGAS protein levels in the chromatin-bound fraction of SK-MEL-2 cells. N.F., non-chromatin-bound fraction; and C.F., chromatin-bound fraction. **M**, **N** Illustrations depicting the establishment of a simple methodology to separate chromatin-bound cGAS by sequential solubilization and elution using RIPA buffer and NaCl. The extracted protein in each fraction number in **M** was used for Western blotting to detect cGAS in **N**. **O** Illustrations showing that the phosphorylated cGAS by an in vitro kinase assay using active RSK2 (left panels) inhibited cGAMP production by in vitro cGAS activity assay using 2′3′-cGAMP ELISA kit.

Based on the previous results that endogenous and stably expressed cGAS and p-RSK2-T577 were mainly detected in the nucleus (Fig. 1) and cGAS and RSK2 are involved in chromatin remodeling [23, 28], we hypothesized that the protein levels of cGAS and RSK2 might correlate in chromatin remodeling. Firstly, we found that the cGAS protein level was increased in RSK2 stably expressing cells compared to mock control cells (Fig. 3A). In contrast, knockdown of RSK2 showed a reduction of cGAS protein level (Fig. 3B). Similarly, RSK2 knockout mouse embryonic fibroblast (MEF) (RSK2^−/−^) showed reduced cGAS protein level compared to that of RSK2^+/+^ MEFs (Fig. 3C). To confirm the cGAS chromatin incorporation, we established a detection strategy of chromatin-bound cGAS. We modified the cytosol and nuclear fractionation strategy using NE-PER nuclear and cytoplasmic extraction reagents (obtained from Thermo-Fisher Scientific). By Western blotting using three fractions (cytosol, nuclear-soluble, and chromatin-bound fractions) (Fig. 3D) that were obtained from cells stably expressing cGAS (Fig. 3E), we found that cGAS was mainly detected in the chromatin-bound fraction (Fig. 3F) and nucleus (Fig. 3G). We further established a simple extraction strategy to separate chromatin-non-bound fraction and chromatin-bound fraction using RIPA buffer containing 1% Triton X-100, 0.1% SDS, 0.5% sodium deoxycholate, 50 mM Tris-HCl pH 7.4, 150 mM NaCl, and 2 mM EDTA (Fig. 3H). Using the cells stably expressing HA-RSK2 (Fig. 3I), we confirmed that the chromatin-bound cGAS was dramatically increased by RSK2 stable overexpression compared to that of mock control cells (Fig. 3J). The ICF result from RSK2 stable cells supported the increase of cGAS nuclear localization (Fig. 3K). In contrast, RSK2 knockdown suppressed the protein level of chromatin-bound cGAS (Fig. 3L). To directly confirm the chromatin tethering of cGAS, we designed cGAS dissociation from chromatin by NaCl treatment using a chromatin pellet in vitro. The obtained nuclear fraction using cytosol and nuclear fractionation kit was dissolved in RIPA buffer, which contained 1% Triton X-100, 0.1% SDS, and 0.5% sodium deoxycholate, to separate the total nuclear protein and chromatin fractions [29]. By Western blotting of the chromatin-bound proteins obtained by 1 M NaCl treatment/elution (Fig. 3M), we found that NaCl-eluted and the rest pellet fractions contained cGAS with high content (Fig. 3N). Importantly, RSK2-mediated cGAS phosphorylation suppressed cGAS activity (Fig. 3O). Taken together, these results demonstrated that RSK2 increases cGAS chromatin incorporation.

### RSK2-mediated cGAS phosphorylation at Ser120 and Thr130 enhances cGAS chromatin incorporation

**Fig. 4:**
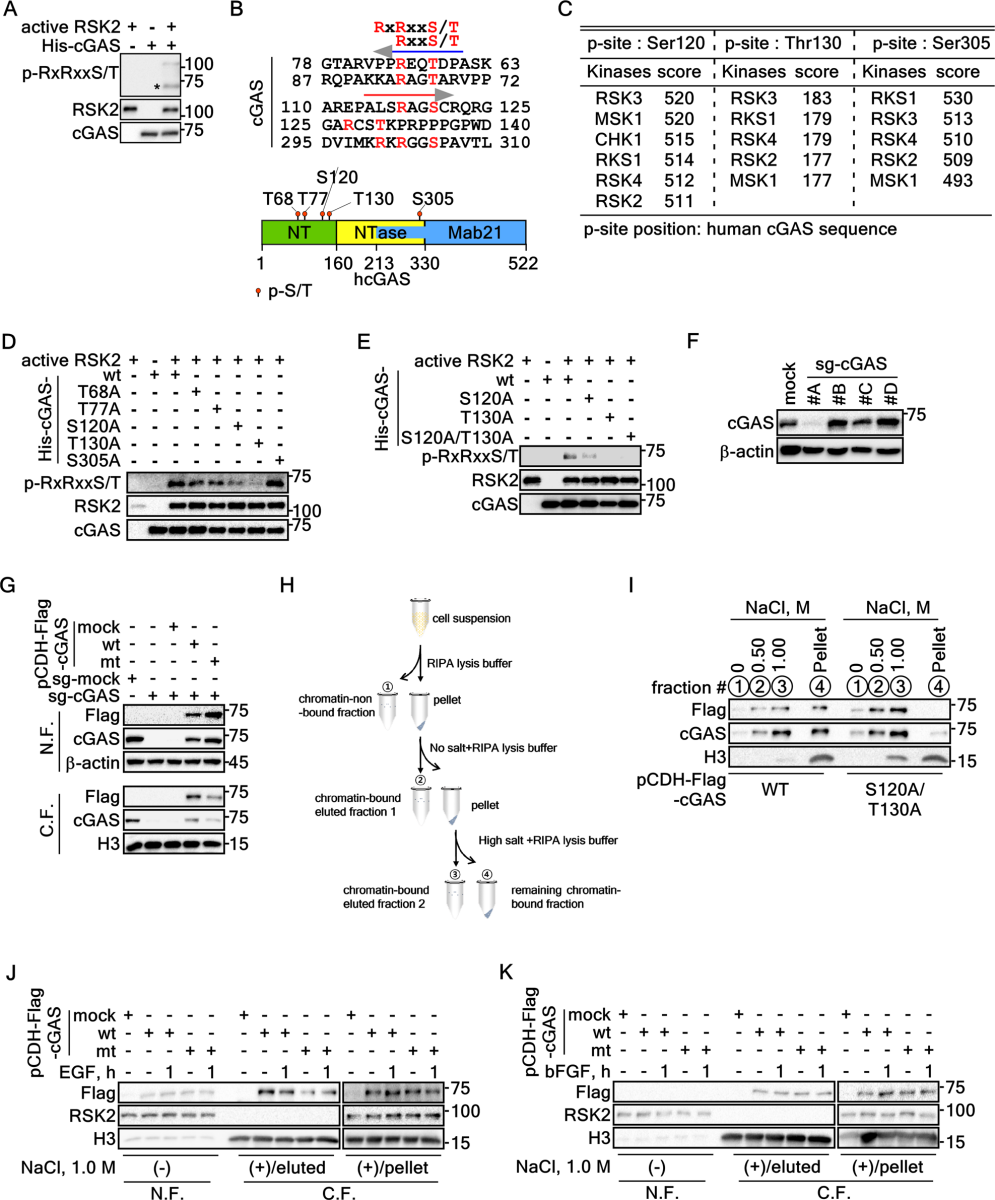
RSK2 phosphorylates cGAS at Ser120 and Thr130. **A** An in vitro kinase assay illustrating that RSK2 phosphorylate cGAS, which is purified from E. coli. The purification processes of cGAS in E. coli are provided in supplementary Fig. S3A and B. **B** Illustrations showing that amino acid analysis to identify potential RSK2 target phosphorylation sites, RxRxxS/T or RxxS/T, in cGAS protein. **C** Summary illustrating the prediction of potential kinases phosphorylating Ser120, The130, and Ser305 of cGAS using PhosphoSitePlus and the Group-based prediction system (GPS 6.0; https://github.com/BioCUCKOO/GPS6.0). Amino acid sequence of cGAS is provided in Supplementary Fig. 1A. Red colors denote the putative RSK2 target phosphorylation sites. **D** An in vitro kinase assay showing that RSK2 phosphorylate cGAS at Ser120 and Thr130 using cGAS-wt and mutant proteins, which are purified from E. coli. The purification processes of cGAS in E. coli are provided in Supplementary Fig. 3C and E. **E** An in vitro kinase assay confirming that phosphorylation target amino acids of cGAS by RSK2 using cGAS-wt, -S120A, -T130A, and -S120A/T130A proteins, which are purified from E. coli. The purification processes of cGAS in E. coli are provided in Supplementary Fig. 3E and F. **F** Western blots illustrating the establishment of cGAS depleting SK-MEL-2 cells by CRISPR/Cas9 small guide RNA for the cGAS (sg-cGAS). The #A-D indicates the clone number of small guide RNA utilized. **G** Western blots illustrating that cGAS-S120A/130A mutant is less incorporated into chromatin compared to that of cGAS-wt by cGAS rescue experiment in SK-MEL-2/sg-cGAS cells. N.F., non-chromatin-bound fraction; C.F., chromatin-bound fraction. **H** Illustration depicting the extraction strategy of cGAS elution from chromatin fraction. This strategy is to determine how strongly the cGAS protein binds to chromatin. The eluted cGAS proteins from chromatin are confirmed by adding of NaCl as shown. N.F., non-chromatin-bound fraction; C.F., chromatin-bound fraction. **I** Western blots illustrating that cGAS phosphorylation by RSK2 enhances cGAS chromatin incorporation by comparison of cGAS proteins of chromatin-released and -bound using NaCl. **J**, **K** Western blots illustrate that EGF- (**J**) or bFGF-induced (**K**) cGAS incorporation into chromatin is abrogated by mutation at Ser120 and Thr130 to Ala. N.F. non-chromatin-bound fraction; C.F. chromatin-bound fraction.

Our previous findings demonstrated that RSK2, a serine/threonine kinase [15], interact with cGAS. Furthermore, we observed that RSK2 promotes the incorporation of cGAS into chromatin (Fig. 3). These observations suggest that RSK2 might directly phosphorylate cGAS. Our in vitro kinase assay using commercially active RSK2 and cGAS purified from E. coli (Supplementary Fig. 3A, B) demonstrated that RSK2 phosphorylated cGAS (Fig. 4A). Amino acid sequence analysis of human cGAS (Supplementary Fig. 1A) suggested that there are five potential phosphorylation sites, composing with RxRxxS/T or RxxS/T [30], at Thr68, Thr77, Ser120, Thr130, and Ser305, for RSK2 (Fig. 4B). Interestingly, phosphorylation site prediction using PhosphoSitePlus (https://www.phosphosite.org/homeAction.action) and the Group-based prediction system (https://github.com/BioCUCKOO/GPS6.0) suggested RSK family members as possible kinases (Fig. 4C). To confirm the phosphorylation target sites of cGAS for the RSK2, we re-purified cGAS proteins that harbored a replaced amino acid to Ala at Thr68, Thr77, Ser120, Thr130, or Ser305 (designated as His-cGAS-T68A, -T77A, -S120A, -T130A, or -S305A) (Supplementary Fig. 3C, D). An in vitro kinase assay using active RSK2 and each of cGAS mutant proteins demonstrated that His-cGAS-S120A and -T130A showed a dramatic reduction of phosphorylation band intensity compared to His-cGAS-wildtype (wt), -T69A, -T77A, and S305A (Fig. 4D). Notably, a double mutation at Ser120 and Thr130 to Ala (Supplementary Fig. 3E, F) totally abrogated the phosphorylation by active RSK2 in in vitro kinase assay (Fig. 4E). The cGAS knockout cells using CRISPR/Cas9 small guide RNA (sg-RNA) (Fig. 4F) were utilized for the rescue the cGAS protein by re-introduction of cGAS-wt or cGAS-S120A/T130A expression vectors (Fig. 4G). We found that the chromatin-bound cGAS-S120A/T130A protein level was decreased compared to that of cGAS-wt, while chromatin-non-bound cGAS and cGAS-wt proteins were vice versa (Fig. 4G). By increasing NaCl concentration in RIPA-pellet fraction (Fig. 4H), the remaining chromatin-bound cGAS-wt protein was higher than that of cGAS-S120A/T130A protein levels (Fig. 4I). This observation suggests that cGAS-wt exhibits a stronger affinity for chromatin under high salt conditions compared with cGAS-S120A/T130A mutant. Furthermore, we found that the treatment of tumor promoters, such as EGF or bFGF, shows a higher level of chromatin association of cGAS compared to the cGAS-S120A/T130A mutant, while the eluted cGAS from chromatin by NaCl addition was vice versa compared to remaining pellet fraction (Fig. 4J, K). Taken together, these results indicated that RSK2-mediated cGAS phosphorylation at Ser120 and Thr130 by tumor promoter stimulation increases cGAS chromatin incorporation.

### Prediction of RSK2 and cGAS signaling pathway in melanoma development

**Fig. 5:**
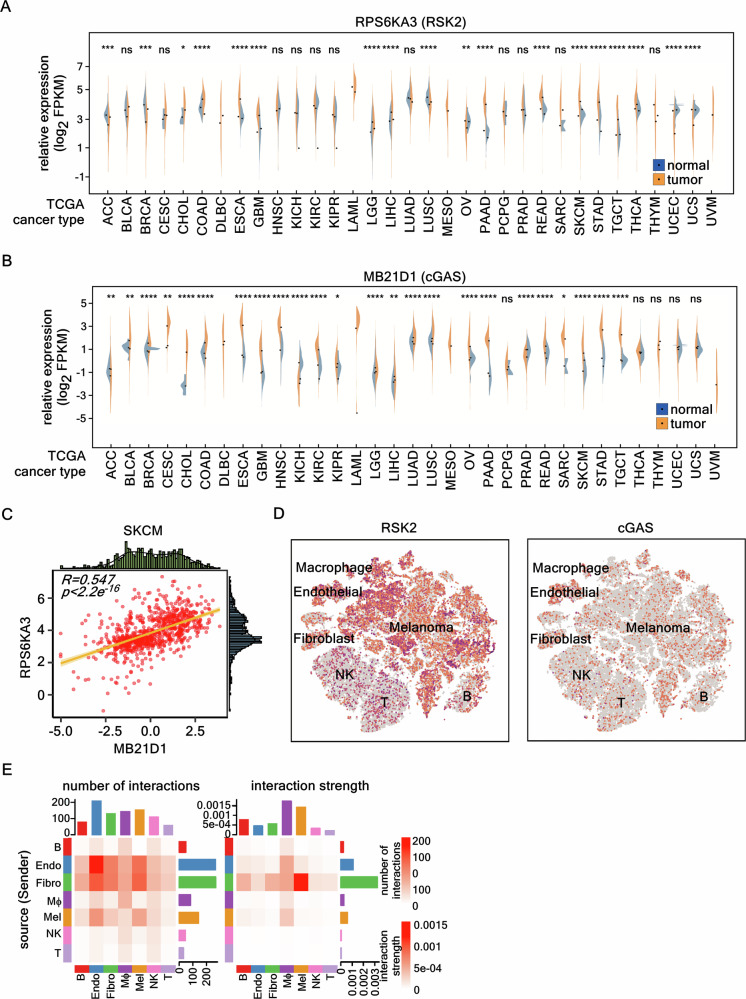
Bioinformatics analysis revealing a positive correlation between cGAS and RSK2 expression in melanoma development. **A** Differential gene expression analysis showing a significant upregulation of RPS6KA3/RSK2 in skin cutaneous melanoma (SKCM) tumor samples compared to normal samples from the TCGA dataset. **B** Differential gene expression analysis showing a significant upregulation of MB21D1/cGAS in SKCM tumor samples compared to normal samples from the TCGA dataset. **C** Pearson correlation analysis of RPS6KA3/RSK2 and MB21D1/cGAS expression. TCGA dataset was used to analysis the correlation of RSK2 and cGAS expression in normal and SKCM tumor samples. *R* = 0.547, *p* < 0.001. **D** Single-cell RNA sequencing analysis. The datasets from Melanoma Institute used to analysis of the expression abundance in melanoma cells. The datasets are annotated to even cell types: T, T cells (CD3D, CD3E); B, B cells (MS4A1, CD79A); NK, NK cells (FGFBP2, KLRD1); MΦ, macrophages (LYZ, CD68, CD14); Mel, melanoma cells (MLANA, PMEL, MITF, DCT); Endo, endothelial cells (VWF, PECAM1); and Fibro, fibroblast cells (COL1A1, COL3A1). **E** CellChat analysis illustrating the heatmap of **D**. The interaction strengths between ligands and receptors indicate that the darker color denotes stronger interaction. **A**, **B** The Wilcoxon test was used for non-parametric significance testing between two groups. ns, *p* > 0.05; *, *p* < 0.05; **, *p* < 0.01; ***, *p* < 0.001; ****, *p* < 0.0001.

Our results indicated that RSK2-mediated cGAS phosphorylation enhanced cGAS chromatin incorporation (Figs. 3 and 4). Since RSK2 plays a key role in cell transformation and cancer development in melanoma [22], deciphering the physiological linkage of RSK2-cGAS signaling in carcinogenesis and cancer cell proliferation is important. Commonly, RSK2 and cGAS strongly involves in the chromatin remodeling that affects gene expression involved in DNA repair, cell cycle regulation, and NF-κB-mediated inflammation and immune responses [31], which act as important factors in cancer development. In initial survey for the content of cGAS in various tissues and cell lines, cGAS proteins were similarly highly detected in every tissue, except ovary, smooth muscle, and adipose tissue, while cGAS mRNAs were highly detected in immune tissues compared to the rest tissues, indicating that cGAS protein content is not restricted to immune cells, but ubiquitously expressed in all organ tissues (Supplementary Fig. 4A, B). Moreover, cGAS protein also highly detected in cancer cells and stem and immune cells (Fig. 4C), indicating that cGAS might play important roles in not only immune response, but also in unknown functions, such as chromatin remodeling. In initial survey of bioinformatics datasets for the pan-cancer mRNA expression profiles obtained from the TGCA-GTEx dataset, we found RSK2 and cGAS mRNA levels were upregulated in skin cutaneous melanoma (SKCM) (Fig. 5A, B). Statistical analysis using the Pearson correlation coefficient demonstrated that gene expression between RSK2 and cGAS showed a significant positive correlation with *R* = 0.547 (*p* < 0.001) in both normal skin and SKCM tumor groups (Fig. 5C, Supplementary Fig. 5A). Since single-cell RNA-seq analysis, which uses expression profiles of all cells within a tissue, is a useful strategy to overlook the heterogeneity of different cell subpopulations within the tissue and the specificity of individual cells for the association [32], we selected seven different datasets (GSM6622292, GSM6622293, GSM6622294, GSM6622296, GSM6622299, GSM6622300, and GSM6622301), which include 19,200 genes from 48,638 cells. From these datasets, the 48,638 cells were annotated into seven cell types, including immune cells, which include T cells (CD3D, CD3E), B cells (MS4A1, CD79A), NK cells (FGFBP2, KLRD1), and macrophages (LYZ, CD68, CD14), and non-immune cells, which include melanoma cells (MLANA, PMEL, MITF, DCT), endothelial cells (VWF, PECAM1), and fibroblast cells (COL1A1, COL3A1) (Fig. 5D, Supplementary Fig. 5B). The results indicated that RPS6KA3/RSK2 has the highest expression abundance in melanoma cells. The statistical analysis indicated that RSK2 association in melanoma cells in the tissues was about 36.4%. Moreover, in RSK2 and cGAS association analysis, we found that cGAS and RSK2 association was observed in 6.23% among 36.4% of RSK2-associated melanoma cells (Fig. 5D). We considered that, if the frequency of the cGAS protein, instead of mRNA, in omics analysis, were used, the association rate between RSK2 and cGAS in melanoma tissues may higher than the 6.23%. Interestingly, CellChat analysis revealed a relatively low number and strength of ligand-receptor interactions between melanoma cells and immune cells, particularly NK and T cells (Fig. 5E), indicating limited communication between these cell types. Taken together with Fig. 1H, these observations suggest a potential interaction between RSK2 and cGAS within the melanoma cell population, potentially contributing to cell transformation and cancer cell proliferation.

### RSK2-mediated cGAS chromatin incorporation is indispensable for tumor promoter-induced cell transformation in JB6 Cl41 and colony growth in melanoma cells

**Fig. 6:**
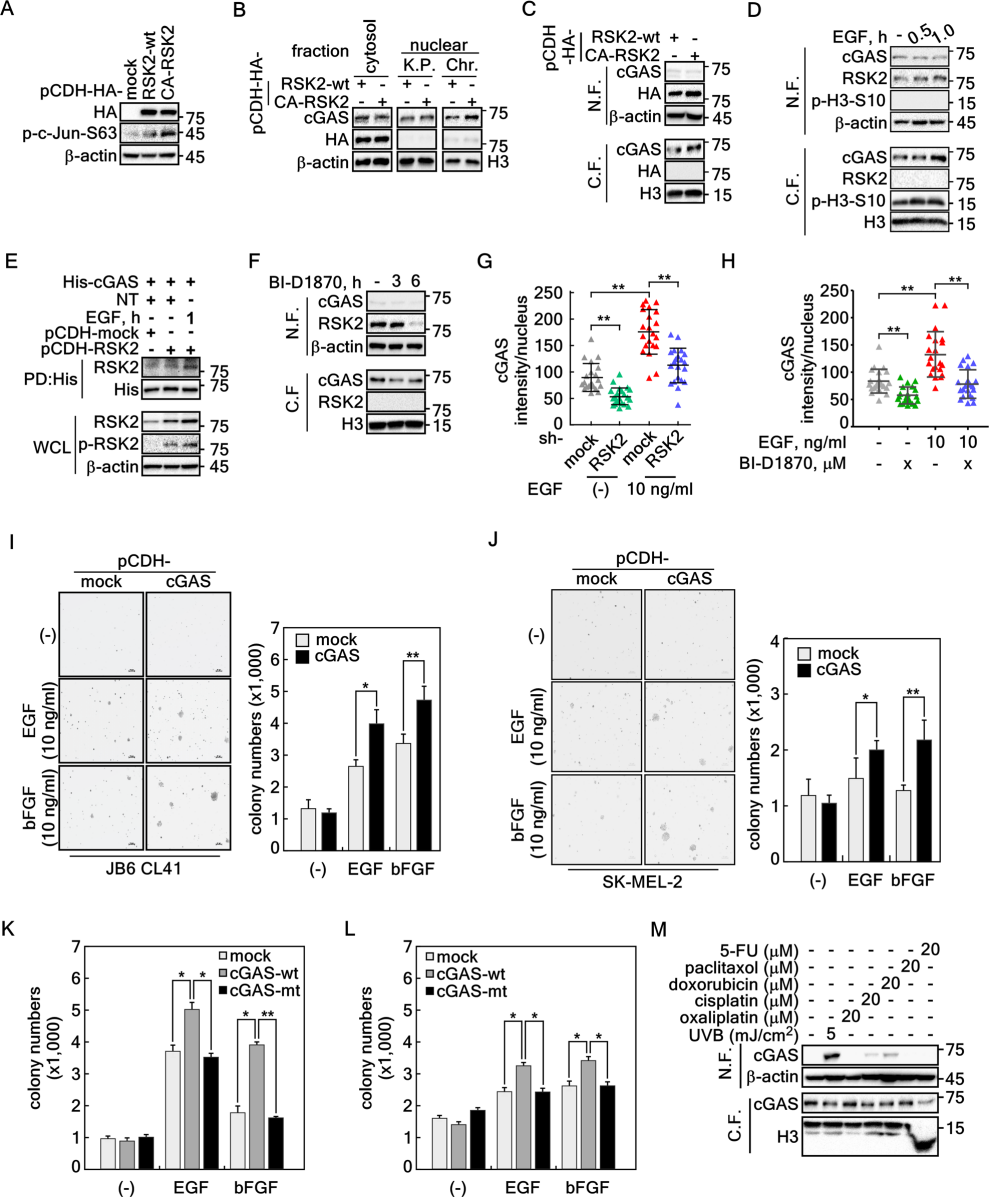
RSK2-mediated chromatin incorporation of cGAS is indispensable for EGF-induced cell transformation and cancer cell colony growth. **A** Western blots illustrating the stable overexpression of RSK2-wt and constitutive active (CA)-RSK2 in SK-MEL-2 cells. **B** Western blots illustrate that CA-RSK2 expression increases endogenous cGAS chromatin tethering in SK-MEL-2 cells. The protein extraction strategy shown in Fig. 3D. was applied. **C** Western blots confirming that CA-RSK2 increases cGAS incorporation into chromatin compared to that of RSK2-wt in SK-MEL-2 cells. The protein extraction strategy shown in Fig. 3H was applied. N.F, non-chromatin-bound fraction; and C.F., chromatin-bound fraction. **D** Western blots illustrating that EGF stimulation increased cGAS chromatin incorporation in SK-MEL-2 cells. The protein extraction strategy as shown in Fig. 3H was utilized. N.F, non-chromatin-bound fraction; and C.F., chromatin-bound fraction. **E** Pull-down/Western blots illustrating that EGF stimulation increased RSK2 and cGAS interaction in SK-MEL-2 cells. **F** Western blots illustrating that RSK2 activity inhibition using BI-D1870 suppresses cGAS chromatin incorporation. The protein extraction strategy shown in Fig. 3H was applied. N.F non-chromatin-bound fraction, C.F. chromatin-bound fraction. **G** Graphs obtained by ICF analysis illustrating that cGAS protein intensity in the nucleus depends on the RSK2 activity. RSK2 knockdown decreases the nuclear intensity of endogenous cGAS in both EGF-untreated and -treated condition. **H** Graphs obtained by ICF analysis illustrating that cGAS protein intensity in the nucleus is is decreased by the inhibition of RSK2 activity. RSK2 activity inhibition by BI-D1870 decreases the nuclear intensity of endogenous cGAS in both EGF-untreated and -treated condition. **I** Illustration showing that cGAS overexpression induces EGF- or bFGF-induced cell transformation in JB6 Cl41 cells. Left panels, Photographs showing the colony formation of JB6 Cl41 cells formed by EGF or bFGF stimulation in soft agar assay. *Graphs*, The colony numbers formed with the bigger size of the criteria in soft agar assay were automatically quantified using ECLIPSE Ti inverted microscope equipped NIS-Elements AR (ver. 4.0) computer software program. **J** Illustration showing that cGAS overexpression induces EGF- or bFGF-induced colony growth of SK-MEL-2 malignant melanoma cells. Left panels, Photographs showing the colony growth of SK-MEL-2 cells by EGF or bFGF stimulation in soft agar assay. *Graphs*, The colony numbers grown with the bigger size of the criteria in soft agar assay were automatically quantified using ECLIPSE Ti inverted microscope equipped NIS-Elements AR (ver. 4.0) computer software program. **K** Graphs illustrating the effect of RSK2-mediated cGAS phosphorylation on the EGF- or bFGF-induced cell transformation in JB6 Cl41 cells. The colony numbers formed with the bigger size of the criteria in soft agar assay were automatically quantified using ECLIPSE Ti inverted microscope equipped NIS-Elements AR (ver. 4.0) computer software program. The Western blots for the confirmation of cGAS expression and photographs for soft agar assay are provided in Supplementary Fig. 6E. **L** Graphs illustrating the effect of RSK2-mediated SK-MEL-2 colony growth induced by EGF or bFGF in soft agar. The colony numbers formed with the bigger size of the criteria in soft agar assay were automatically quantified using ECLIPSE Ti inverted microscope equipped NIS Elements AR (V. 4.0) computer software program. The Western blots for the confirmation of cGAS expression and photographs for soft agar assay are provided in Supplementary Fig. 6F. **M** Western blots illustrate that the released and tethered cGAS protein levels between non-chromatin-bound and chromatin-bound forms are inversely correlated. Histone H3 in C.F. and β-actin were used as an internal control for equal protein loading. **G**–**L** Error bars represent mean ± standard error of the mean (SEM). *, *p* < 0.05; **, *p* < 0.01 (Student’s *t* test).

Our previous results demonstrated that RSK2 plays a key role in cell transformation induced by growth factors, such as EGF [33, 34] and bFGF [35], and cancer cell proliferation [25]. In this process, RSK2 also participates in chromatin remodeling by inducing phosphorylation of histone H3 and H2B [18, 26]. Since RSK2 induced cGAS chromatin incorporation (Figs. 3 and 4) by phosphorylation at Ser120 and Thr130 (Fig. 4), we hypothesized that RSK2-cGAS signaling pathway might play an essential role in tumor promoter-induced cell transformation and cancer cell proliferation. To elucidate this hypothesis, we established SK-MEL-2 cells stably expressing RSK2-wt or constitutively active (CA)-RSK2 (Fig. 6A). The effect of CA-RSK2 was confirmed by the induction of phosphorylation of c-Jun at Ser63 (Fig. 6A) as previously shown [36]. Furthermore, while stable overexpression of CA-RSK2 did not increase the cytosolic cGAS protein levels, it increased the total nuclear cGAS protein levels (karyoplasmic + chromatin-bound of cGAS in RSK2-wt *vs*. CA-RSK2) (Fig. 6B). More interestingly, chromatin-bound cGAS levels were dramatically increased by CA-RSK2 overexpression, while karyoplasmic (the soluble fraction of the nuclear fraction) cGAS protein levels were not changed (Fig. 6B), indicating that RSK2 activity might induce cGAS chromatin incorporation. Using the same strategy for cGAS chromatin binding as shown in previous results (Figs. 1, 2 and 4), we confirmed that the increased cGAS protein was detected in chromatin fraction when the cell lysates were obtained by the RIPA extraction (Fig. 6C). Importantly, we confirmed that EGF increased chromatin-bound cGAS and p-H3-S10 protein levels (Fig. 6D), suggesting that the RSK2-cGAS signaling might increase cGAS chromatin binding. These results were strongly supported by ex vivo pull-down assay using bacterial expressed His-cGAS and the cell lysates obtained from RSK2 stably expressing cells presence of either EGF stimulation or not. The results demonstrated that interaction between cGAS and RSK2 was increased in RSK2-overexpressing and EGF-stimulated cells compared to mock and RSK2-overexpressing cells without EGF stimulation (Fig. 6E). Notably, RSK2 inhibition by BI-D1870 treatment suppressed cGAS chromatin binding (Fig. 6F). These results demonstrated that RSK2 activity is indispensable for cGAS chromatin incorporation. Notably, RSK2 knockdown decreased cGAS nuclear intensity as observed by ICF assay (Fig. 6G, Supplementary Fig. 6A). Importantly, the increased nuclear cGAS by EGF stimulation in mock was significantly inhibited by RSK2 knockdown (Fig. 6G, Supplementary Fig. 6A). Similar results obtained by RSK2 knockdown were observed when the cells were treated with BI-D1870 (Fig. 6H, Supplementary Fig. 6B). These results demonstrated that cGAS chromatin incorporation requires RSK2 activity induced by growth factor stimulation. To analyse the RSK2-cGAS signaling pathway in cell transformation, we established JB6 Cl41 and SK-MEL-2 malignant melanoma cells stably overexpressing cGAS (Supplementary Fig. 6C, D). Using these cells, we conducted anchorage-independent cell transformation and cancer cell colony growth assays induced by EGF or basic fibroblast growth factor (bFGF). We confirmed that EGF stimulation increased colony formation in soft agar by EGF or bFGF stimulation in JB6 Cl41 cells (Fig. 6I). Moreover, we also confirmed that colony growth of malignant melanoma cells in anchorage-independent condition was dramatically increased in the stably cGAS overexpressing cells by EGF or bFGF stimulation (Fig. 6J). The biological effect of cGAS phosphorylation mediated by RSK2 was confirmed by cell transformation activity of JB6 Cl41 cells and colony growth activity of SK-MEL-2 cells by overexpression of cGAS-wt or cGAS-S120A/T130A (designated cGAS-mt). The results demonstrated that EGF or bFGF-induced increased cell transformation activity in cGAS-wt overexpression was abrogated by cGAS-S120A/T130A overexpression in JB6 Cl41 cells (Fig. 6K, Supplementary Fig. 6E). Similarly, colony growth of SK-MEL-2 cells overexpressing cGAS-S120A/T130A in soft agar was decreased compared to cGAS-wt overexpressing SK-MEL-2 cells (Fig. 6L, Supplementary Fig. 6F). The RSK2-cGAS stable coexpression showed the increase of S-phase cell population and the decrease of G_1_-phase cell population compared to cGAS alone or RSK2 alone (Supplementary Fig. 6G), indicating that cell proliferation is increased as shown in previous [33]. Additionally, the released cGAS tethered in chromatin was increased by the stimuli that directly induced DNA, such as UVB, cisplatin, and doxorubicin (Fig. 6M), indicating that the role of cGAS in chromatin remodeling is differently regulated depending on the different stimuli. Taken together, these results demonstrated that RSK2-cGAS signaling pathway plays an important role in growth factor-induced cell transformation and cancer cell colony growth.

## Discussion

Since RSK2 phosphorylates CREB [37], histone H3 [17], and histone acetyltransferase (HAT) [38], the physiological association of RSK2 in chromatin remodeling is well recognized. When cells are in a quiescent stage, unphosphorylated RSK2 that is bound with CBP is dissociated by PDK1-mediated phosphorylation at Ser227, resulting in activation of RSK2 [23]. The activated RSK2 phosphorylates histone H3 at Ser10 [33] and CREB [23, 37]. At the same time, the dissociation of CBP from RSK2 allows for an increase of HAT activity, resulting in histone H3 acetylation at Lys14 [23]. Cells obtained from Coffin-Lowry syndrome (CLS) patients, caused by the deletion, nonsense, and missense mutations of RSK2, show defects in EGF-induced histone H3 phosphorylation, and introduction of RSK2-wt into CLS patient cells restores p-H3-S10 protein levels [16]. Moreover, RSK2 phosphorylates H2B at Ser32 and H2AX at Ser139 [19], indicating that nuclear RSK2 is strongly involved in chromatin remodeling [39]. Although RSK2-mediated histone phosphorylation is not an absolute condition for chromatin remodeling, it is clear that RSK2-mediated phosphorylation of epigenetic factors can modulate gene expression that is mediated by chromatin remodeling. Because of these reasons, CLS has classified as a type of chromatin disease [39]. In this study, we found the surprising fact that cGAS localization was differentially detected depending on the methodologies, transient expression and endogenous and stable expression (Fig. 1). cGAS binding to chromatin was recently identified based on its DNA-binding properties [40], suggesting that cGAS may be redistributed to the cytosol during the resolution of cell division and the reformation of the nuclear envelope [40]. To detect the strong association of cGAS with chromatin, we used a RIPA extraction buffer containing 1% Triton X-100, 0.1% SDS, 0.5% sodium deoxycholate, 50 mM Tris-HCl (pH 7.4), 150 mM NaCl, and 2 mM EDTA, following our previous protocol [29]. With this RIPA buffer, we solubilized the nuclear pellet fraction, which was obtained through centrifugation after nuclear membrane disruption. Since this nuclear pellet fraction contains chromatin, nuclear lamina, and nuclear envelope components, detecting cGAS in this fraction indicates that cGAS is predominantly associated with chromatin DNA. Importantly, we observed that cGAS incorporation into chromatin is dependent on RSK2 (Fig. 4I–K). Moreover, mutating Ser120 and Thr130 to Ala in cGAS suppressed its incorporation into chromatin (Fig. 4G–I). These results demonstrated that RSK2-mediated cGAS phosphorylation directly affected cGAS’s chromatin incorporation. However, the detailed molecular mechanisms of the structural basis have not been elucidated.

cGAS primarily engages the nucleosome core particle (NCP) through binding to the so-called acidic patch composed of Glu61, Asp90, Glu92, which are interacted with Arg236, Lys254, Arg255 in DNA binding B-site via electrostatic interaction [41]. Substitutions of either arginine residues on cGAS or key acidic patch residues in the H2A-H2B dimer completely abolish the binding of cGAS to the nucleosome and unleash cGAS activity on chromatinized DNA [42]. In addition, a second interface is contributed by additional B-site residues that can interact with histones H2A and H2B and nucleosomal DNA at superhelical location (SHL) position 5.5/6, stabilizing a more rigid conformation of the cGAS:NCP complex [43]. In examination for the structure determination using human cGAS, intriguing discovery was obtained by which cGAS interacted with a secondary nucleosome in trans, leading to the formation of a 2:2 cGAS:NCP complex and even higher ordered cGAS:NCP oligomers [41, 42, 44]. However, no reports have mentioned the molecular mechanism how growth factor-mediated cGAS chromatin incorporation occurs. In this study, we provided evidence that cGAS activity is regulated by phosphorylation at the N-terminal unstructured domain. The results clearly showed that the stimulation of tumor promoters, such as EGF and bFGF, increased anchorage-independent cell transformation and cancer cell colony growth in cGAS-wt overexpressing cells compared to that of cGAS-mt overexpressing cells (Fig. 6, Supplementary Fig. 6). Interestingly, B-lymphoid tyrosine kinase-mediated cGAS phosphorylation at Tyr215 promotes nuclear accumulation of cGAS by etoposide treatment [14]. Moreover, cGAS knockdown inhibits tumor growth in in vivo mouse model using non-small cell lung carcinoma, indicating that cGAS promotes tumorigenesis [14]. Since DNA damage and growth factor stimulation induce dynamic chromatin structure change, we currently believe that cGAS might participate in chromatin remodelling in not only cell division but also hetero-euchromatin remodelling, depending on the cellular context.

Chromatin remodeling within the nucleus is a dynamic process critical for regulating gene expression and cell division, particularly in the nuclear envelope (NE). The regulation of nuclear membrane integrity plays key roles in processes such as chromatin organization, DNA replication [45] and repair [46, 47], gene expression regulation [48, 49], and cell division [50]. When nuclear membrane integrity is compromised, leading to exposure of naked DNA, cGAS activation via DNA sensing may be increased. A recent study showed that barrier-to-autointegration factor (BAF) antagonizes cGAS activity on genomic self-DNA [11], underscoring the close relationship between cGAS activity regulation and chromatin remodeling. Importantly, the N-terminal unstructured region of cGAS (aa 1–160) is highly phosphorylated during mitosis, especially in the G_2_/M phase [51]. Additionally, cGAS activity inhibition is positively correlated with cGAS phosphorylation and chromatin tethering [51]. Moreover, as RSK2 is known to phosphorylate histone H3 at Ser10 [17, 33], its involvement in chromatin remodeling is well established [39]. Previous results indicated that, when RSK2 was overexpressed, it arrested cells in the S phase and dramatically increased the G_2_/M population under serum starvation (0.1% FBS) followed by EGF stimulation [26, 33]. This result suggests that RSK2 activation sensitizes cells to progress through the G1/S phase when stimulated by low concentrations of mitogen. After exposure to 10% FBS, there was a dramatic transition to the G_2_/M phase, indicating that the RSK2 signaling pathway plays a crucial role in G_2_/M phase transition. At the same time, phosphorylated p53 at Ser15 was also increased. Based on this rationale, we believe that cGAS phosphorylation might participate chromatin remodeling via unknown mechanisms. However, molecular mechanisms for the cGAS involvement in chromatin remodeling have not been elucidated. This study provided evidence that cGAS phosphorylation by RSK2 is required to interact with chromatin DNA, resulting in growth factor-induced cell transformation and colony growth of cancer cells.

**Fig. 7:**
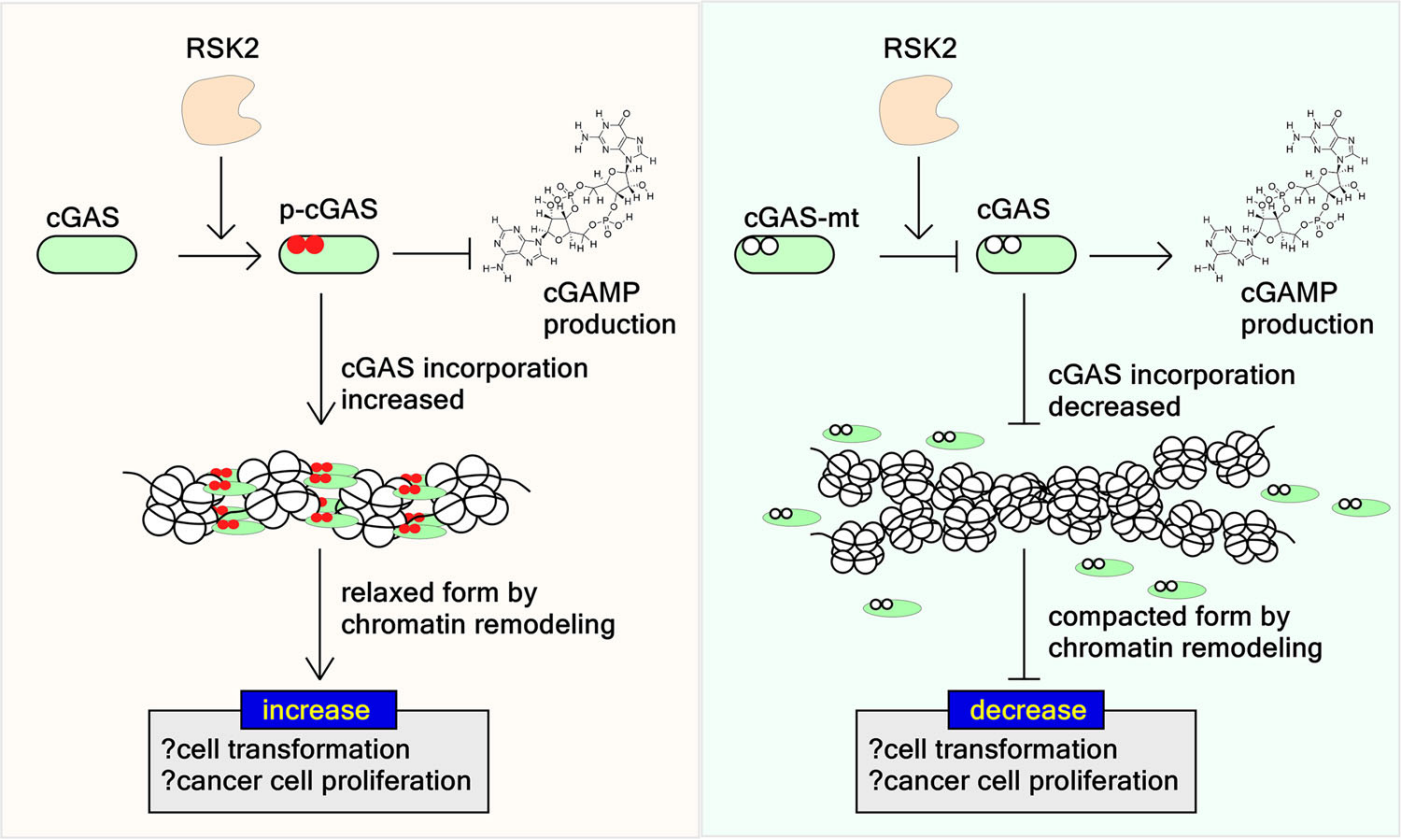
Proposed mechanism for the RSK2-mediated cGAS phosphorylation and chromatin remodeling. RSK2 activated by tumor promoters, such as EGF and bFGF, phosphorylate cGAS at the nucleus at Ser120 and Thr130. The phosphorylation of cGAS increases the incorporation into chromatin, which may result in the enhancement of a relaxed form of chromatin structure. In this situation, cells are generally activated, resulting in cell transformation of normal cells and cancer cell proliferation. At the same time, phosphorylated cGAS by RSK2 suppressed their activity, resulting in inhibition of cGAMP production. However, cGAS mutant at Ser120 and Thr130 to alanine showed reverse phenomenon in cell transformation, cancer cell proliferation, and cGAMP production.

Previous studies have shown that treatment with etoposide, an anticancer drug, induces phosphorylation of cGAS at Ser120 and Ser305 by CHK2 [52]. Interestingly, the efficacy of cGAS-mediated L1 retrotransposition varied depending on the mutation of these phosphorylation sites to alanine. Furthermore, CHK2-mediated phosphorylation of cGAS at Ser120 and Ser305 enhanced the recruitment of TRIM41 to ORF2p, leading to increased ORF2p ubiquitination and degradation [52]. Phosphorylation-mimetic mutations to glutamate further enhanced cGAS association with TRIM41. These findings suggest that the CHK2-cGAS-TRIM41-ORF2p axis represents a novel mechanism for modulating aging and tumorigenesis [52]. We currently suggest that phospho-mimetic mutants of cGAS might promise the decipher detailed molecular mechanisms of how the RSK2-cGAS axis regulates chromatin remodeling at the INM. We suggest that RSK2-mediated phosphorylation of cGAS may influence nucleosome status, potentially regulating the transition between compacted and relaxed chromatin forms, which is critical for heterochromatin/euchromatin dynamics at the G_2_/M cell cycle checkpoint. Our results demonstrated that G_2_/M arrest increased the levels of cGAS in both free and chromatin-incorporated forms (Fig. 1G). More significantly, RSK2-mediated cGAS phosphorylation at Ser120 and Thr130 enhanced its incorporation into chromatin (Fig. 4G–K). These findings suggest that RSK2-mediated phosphorylation may suppress cGAS activity. This hypothesis is supported by evidence that cGAMP, the product of cGAS, suppresses cancer cell proliferation and increases anticancer activity [53, 54]. We also confirmed that RSK2-mediated phosphorylated cGAS by active RSK2 suppressed cGAMP production in vitro (Fig. 3O). These results suggested a linkage of the molecular mechanisms between cGAS phosphorylation-mediated activity regulation and chromatin tethering. As a summary of this research, we proposed that active RSK2 phosphorylates cGAS at Ser120 and Thr130 by tumor promoters, such as EGF and bFGF. The phosphorylated cGAS can involve in chromatin remodeling by incorporation into chromatin, resulting in the formation of relaxed form of chromatin, resulting in increase of cell transformation and cancer cell proliferation. At the same time, phosphorylation of cGAS by RSK2 inhibits cGAS activity to produce cGAMP, which induces death-inducing cytokine production, resulting in the inhibition of cell proliferation (Fig. 7).

## Materials and methods

### Reagents

Reagents for molecular and cellular biological studies, including EGF (cat #: C2211) and BI-D1870, were purchased from Sigma-Aldrich (Sigma‒Aldrich Korea, Gangnam, Seoul, Korea). Basic fibroblast growth factor (bFGF, cat #: S6999) was purchased from Selleck Chemicals (Houston, TX, USA). Antibodies used for Western blotting, immunoprecipitation (IP), and immunocytofluorescence (ICF), including anti-RSK2 (cat #: sc-9986), anti-p-RSK2-577 (cat #: sc-16407), anti-HA (cat #: 901501), anti-p-RSK2-T359/S363 (cat #: sc-9344), anti-γH2AX (cat #: sc-517348), anti-Lamin B (cat #: sc-6216), and anti-β-actin (cat #: sc-47778), were obtained from Santa Cruz Biotechnology (Dallas, TX, USA). Anti-cGAS (cat #: 15102), anti-Histone H3(cat #: 4499), anti-p-Histone H3(S10) (cat #: 53348), anti-RXRXXS/T (cat #: 10001), anti-p-c-Jun-ser63 (cat #: 9165) were obtained from Cell Signaling Technology (Koram Biotech Corp., Gangnam, Seoul, Korea). Anti-DDDDK-tag (as known as Flag) (Cat #: M185), anti-DDDDK-tag-HRP (as known as Flag-HRP, Cat #: M185-7), anti-His-HRP (Cat #: D291-7) and anti-HA-HRP (Cat #: M180-7) antibodies were obtained from MBL International Corporation (Woburn, Massachusetts, USA). Anti-Xpress (Cat #: 46-0528) was obtained from Invitrogen. Protein G Sepharose beads (Cat #: 17-0618-02) were purchased from GE Healthcare (Chicago, Illinois, USA). HisPur™ Ni-NTA resin (Cat #: 88221) was obtained from Thermo-Fisher Scientific (Waltham, Massachusetts, USA).

### Processing and integration of scRNA-seq data

The open microarray dataset GSE215120 was obtained from the Gene Expression Omnibus database (GEO, https://www.NCBAppeal.nominated.nih.gov/geo) at National Center for Biotechnology Information. We utilized easyTCGA (*v* = 0.0.1.8000) to fetch and combine pan-cancer RNA-seq data from the TCGA and GTEx databases, incorporating clinical sample phenotype information. To visualize the data, we used the Seurat (*v* = 4.9.9) [55] for further analysis. First, we applied the PercentageFeatureSet function to calculate the percentages of genes for red blood cells, mitochondria, and ribosome, and filtered genes and cells based on the specific criteria as following: nFeature > 300, nCount > 50, mitochondrial genes < 15%, ribosomal genes > 10%, and red blood cell genes < 1%. Second, we used the SCTransform (v0.3.5) [56] to standardize the filtered data, perform scaling, and identify highly variable genes. Additionally, we applied the CellCycleScoring function to investigate the influence of the cell cycle on the data. We further used the harmony (v0.1.1) [57] to integrate and reduce the dimensionality of the filtered data. Finally, we performed dimensionality reduction, clustering, and visualization on scRNA-Seq data. Principal component analysis (PCA) is utilized to reduce the dimensionality of the integrated dataset. Based on the first 20 principal components (PCs), the integrated dataset was further simplified into a two-dimensional (2D) space using t-distributed Stochastic Neighbor Embedding (tSNE) with approximations and projections by Uniform Manifold Approximation and Projection (UMAP) for visualization. The functions of FindNeighbors, FindClusters, RunTSNE, and RunUMAP were utilized to execute these analyses with a resolution of 0.6, resulting in 20 identified clusters. The FindAllMarkers function was applied to compute characteristic marker genes for each cluster and to perform cell annotation.

### Cell-Cell communication analysis

CellChat (v1.5) [58] in CellChatDB human database (http://www.cellchat.org/), a curated database of ligand-receptor interactions and signaling pathways, was used to infer cell-cell communication networks based on gene expression data. This tool was employed to identify and quantify potential interactions between overexpressed genes in melanoma cells and their corresponding receptors or ligands expressed by neighboring cells within the tumor microenvironment. In our analysis, we first sorted out overexpressed genes within the melanoma cell population and used CellChat to predict the probability of interaction between these overexpressed genes and their respective receptors or ligands expressed in neighboring cells that composing the SKCM samples.

### Bioinformatic analysis of cGAS protein and mRNA content

The protein content of cGAS in various human tissues was downloaded from the Human Protein Atlas (HPA, https://www.proteinatlas.org/). The protein content of cGAS in various human cell lines was downloaded from ProteomicsDB (https://www.proteomicsdb.org/). This data represents the relative abundance of cGAS protein within different cell lines based on mass spectrometry measurements. The mRNA content of cGAS in various human tissues was downloaded from the Human Protein Atlas (HPA, https://www.proteinatlas.org/). The nTPM indicates the consensus dataset consisted of normalized expression (nTPM, normalized a sum of 1 million transcript).

### Data processing and statistical analysis

*R* (*v* = 4.3.1) was used for data processing and statistical analysis. Non-parametric tests between two groups were conducted using the Wilcoxon rank-sum test. The rcorr function was used to compute the matrix of Pearson’s r rank correlation coefficients for all possible pairs of columns of a matrix, as well as the asymptotic p-values.

### Computational docking of RSK2 and cGAS

Protein-protein docking simulations using Discovery Studio 2021 software (Dassault Systèmes BIOVIA, San Diego, CA, USA). The crystal structures of human cGAS (PDB ID: 4MKP) and RSK2 (PDB ID: 5D9L) retrieved from the Protein Data Bank (https://www.rcsb.org/) were used protein-protein docking using Discovery Studio as described previously [59]. The docking simulations were conducted using the ZDOCK protocol, which employs a fast Fourier transform-based algorithm to explore potential binding modes between the two proteins. This protocol generated a set of potential docking poses ranked according to their predicted binding affinities. The top-ranked poses were then subjected to further refinement using the RDOCK protocol, which optimizes the interaction energy between the proteins through a CHARMM-based energy minimization procedure. This refinement step aimed to identify the most energetically favorable binding modes between RSK2 and cGAS. The resulting docking models were analyzed to identify potential interaction sites and residues involved in the binding interface between the two proteins. We specifically focused on residues predicted to form hydrogen bonds, salt bridges, and hydrophobic interactions, as these types of interactions often play a crucial role in protein-protein complex formation.

### Cell culture

HEK293T, HeLa, and RSK2^+/+^ and RSK2^−/−^ mouse embryonic fibroblast (MEF) cells were cultured in Dulbecco’s modified Eagle’s medium (DMEM, cat #: 10-013-CV; Corning Korea, Seoul, Korea), and SK-MEL-2, SK-MEL-28, and JB6 Cl41 cells were cultured in minimum essential medium supplemented with L-glutamine (MEM, cat #: 10-010-CV; Corning Korea) and 10% fetal bovine serum (FBS, cat#: 35-015-CV; Corning Korea). HT-29 and MH7A cells were cultured in RPMI 1640 medium supplemented with L-glutamine (RPMI 1640, cat #: 10-040-CVRC; Corning Korea). HEK293T, HeLa, SK-MEL-2, SK-MEL-28, JB6 Cl41, HT-29 cells were purchased from ATCC (Koram Biotech Co., Kangnam, Seoul, Korea). MH7A, a human RA synovial cell line obtained from the Riken cell bank (Tsukuba, Ibaraki, Japan). RSK1^+/+^ and RSK2^−/−^ MEFs were generously gifted from Dr. J.C. Brunung, Institute for Genetics, Center for Molecular Medicine Cologne, Cologne, Germany. The cell lines were used at passage<15 and were periodically authenticated by monitoring cell morphology, performing growth curve analysis, and undertaking contamination inspection for contaminants such as mycoplasma. All cells were maintained at 37 °C in a 5% CO_2_ incubator and passaged at approximately 90% confluence.

### Expression vectors

HA and Flag-tag fusion proteins were constructed by basic recombinant DNA technology using pCMV-HA from TAKARA Bio INC. (cat #: 635689, Kusatsu, Shiga, Japan) and pBICEP-CMV-2 Flag from Sigma‒Aldrich (cat #: E0904, Sigma‒Aldrich Korea, Gangnam, Seoul). Mammalian expression vectors for pTRIP-CMV-GFP-FLAG-cGAS (cat #:86675) were purchased from Addgene (Watertown, MA, USA). RSK2 wildtype and its derived constructs and cGAS wildtype and its derived constructs were recombined to pCMV-HA or -Flag-tagged plasmid, respectively. To establish cells stably expressing Flag-cGAS, HA-RSK2, or HA-RSK2-Y707A in SK-MEL-2 cells, the pCDH-CMV-MCS-EF1-puro viral vector from Addgene was used. The expression vectors utilized in this study were confirmed by DNA sequencing before use.

### Western blot and immunoprecipitation

Cell lysates (20 μg) extracted using RIPA cell lysis buffer containing 1% Triton X-100, 0.1% SDS, 0.5% sodium deoxycholate, 50 mM Tris-HCl (pH 7.4), 150 mM NaCl, and 2 mM EDTA were resolved by SDS‒PAGE, transferred onto PVDF membranes, and hybridized with specific antibodies as indicated. For IP, cell lysates (200 μg) were coupled with specific antibodies (2 μg/ml) at 4 °C for 4 h or overnight, combined with 50% of protein G Sepharose slurry (GE Healthcare) for additional 2 h at 4 °C, and precipitated by centrifugation at 1000 rpm for 1 min at 4 °C. The proteins bound to the beads were washed using washing buffer (20 mM Tris at pH 8.0, 100 mM NaCl, 1 mM EDTA, and 0.5% NP-40), boiled, and visualized by Western blotting as described above.

### Viral infection for ectopic expression and gene knockdown

HEK293T cells were used to produce viral particles by co-transfection of a lentiviral expression vector and retroviral packaging system vectors. In brief, viral vectors for overexpression or knockdown were transfected with packaging system vectors into HEK293T cells and cultured for 48 h with a complete growth medium at 37 °C in a 5% CO_2_ incubator. The viral particles were harvested at 48 h by filtration of the culture supernatant using 0.45 μm acetate syringe filters and used for infection into SK-MEL-2 cells by culturing for 24 h in complete medium containing polybrene (final concentration of 1 μg/ml; Sigma‒Aldrich). The cells were then treated with puromycin (2 μg/ml) for 2 days, pooled, and utilized for the following experiments. The efficiency of overexpression or gene knockdown cells was evaluated by Western blotting using specific antibodies as indicated.

### Establishment of cGAS knockout SK-MEL-2 cells

For CRISPR/Cas9 gene targeting, we used a lentiviral system in which a U6 promoter-driven guide RNA and an MND promoter-driven Cas9-T2A-puromycin resistance cassette are constitutively expressed from a single, self-inactivating lentivirus. SK-MEL-2 cells were infected with lentiviral particles and selected with 2 μg/ml puromycin (Thermo-Fisher Scientific) for 2 days. Gene targeting was evaluated by Western blot using an antibody that specifically recognizes the endogenous target protein. The sg-RNA primers targeting cGAS gene are following: cGAS #A, 5′-GAAGTGCGACTCCGCGTTCAG-3′; cGAS #B, 5′-GAAGGCCTGCGCATTCAAAAC-3′; cGAS #C, 5′-GCCTTGTACCCAAGCATGCAA-3′; cGAS #D, 5′-GATCCTTCTCTCACATCGAAA-3′.

### DNA association assay of cGAS

To confirm the DNA association of cGAS, RIPA buffer containing 1% Triton X-100, 0.1% SDS, 0.5% sodium deoxycholate, 50 mM Tris-HCl (pH 7.4), 150 mM NaCl, and 2 mM EDTA was used. Briefly, SK-MEL-2 cells stably or transiently expressing mock or Flag-cGAS were harvested, washed, and suspended in RIPA buffer. The suspension was stand on ice for 30 min, and supernatant soluble protein fraction (soluble fraction, S.F.) was recovered by centrifugation at 12,000 rpm for 4 min at 4 °C. The remaining pellets were suspended in RIPA buffer, sonicated for 15 cycles of 30 seconds at full power and a 30-second resting interval, and centrifuged 12,000 rpm for 5 min at 4 °C to obtain chromatin-bound protein fraction (chromatin fraction, C.F.). Proteins in the S.F. and C.F. were resolved by SDS-PAGE and visualized by Western blotting using a cGAS-specific antibody as indicated.

### Confocal microscopy

The SK-MEL-2 and HEK293T cells (1 × 10^4^ cells/well) that stably or transiently expressed Flag-cGAS or HA-RSK2 were seeded in 4-chamber slides and cultured overnight. The cells were fixed with 4% paraformaldehyde for 15 min at room temperature, permeabilized using 0.5% Triton X-100/1 × PBS, and blocked with 1× PBS/3% BSA at 37 °C for 1 h. To visualize the target proteins, the cells were hybridized with primary antibodies against Anti-DDDDK-tag (as known as Flag) (Cat #: M185, MBL International Corporation), p-RSK2-T577(cat #: sc-16407, Santa Cruz), or cGAS (cat #: 15102, Cell Signaling Technology) overnight at 4°C and then hybridized with secondary antibodies, Alexa Fluor 488-conjugated goat anti-mouse (cat#: A-11029, Invitrogen, Waltham, MA, USA), Alexa Fluor 488-conjugated goat anti-rabbit (cat#: A-11029, Invitrogen), Alexa Fluor 568-conjugated goat anti-rabbit (cat#: A-11031, Invitrogen), or Alexa Fluor 568-conjugated goat anti-goat (cat#: A-21235, Invitrogen), at room temperature for 1 h. The target proteins were observed under an LSM 710 laser scanning confocal microscope (Carl Zeiss Korea Co. Ltd., Seoul, Korea). The fluorescence intensity was measured using NIH Image J computer programs (ver. 1.53a, National Institutes of Health, Bethesda, MD, USA).

### Protein expression and purification

Wildtype and mutant types of the full length of cGAS cDNA were cloned into the pET-28a vector with an N-terminal 6x His tag. The expression of cGAS proteins in Rosetta (DE3) was induced by 0.5 mM of isopropyl β-D-1-thiogalactopyranoside treatment at 20 °C for 20 h. The bacteria pellets were lysed using bacteria lysis buffer (50 mM Tris pH 8.0, 500 mM NaCl, 1 mM 2-Mercaptoethanol, 10% glycerol, protease inhibitor), and cGAS proteins were partially purified by using HisPur Ni-NTA resin (cat #: 88221; Thermo-Fisher Scientific). The resin was washed with washing buffer (50 mM Tris (pH 8.0), 500 mM NaCl, 20 mM imidazole, 1 mM 2-mercaptoethanol, and protease inhibitor cocktail). The purified proteins were confirmed by SDS-PAGE and Western blotting using a cGAS specific antibody.

### In vitro kinase assay

Wildtype and mutant His-cGAS proteins (300 ng) purified from Rosetta (DE3) were subjected to an in vitro kinase assay with active RSK2 (50 ng) and cold ATP (final concentration, 100 μM). The kinase reaction was carried out at 30 °C for 30 min and stopped by adding 6× SDS sample buffer and boiling for 5 min. The phosphorylated cGAS proteins were visualized by 8–10% SDS-PAGE and Western blotting using a phospho-RxRxxpS/pT specific antibody as indicated.

### In vitro pulldown assay

Partial purified His-cGAS fusion proteins bound to HisPur Ni-NTA resin (cat #: 88221, Thermo-Fisher Scientific) were combined with cell lysates obtained from SK-MEL-2 cells stably expressing HA-mock or HA-RSK2 stimulated with or without EGF (10 ng/ml) and gentle rocking overnight at 4 °C. The reaction mixtures were centrifuged to recover the resin and washed with bead washing buffer (100 mM NaCl, 20 mM Tris (pH 8.0), 1 mM EDTA, and 0.5% NP-40). The co-precipitated RSK2 proteins by cGAS pulldown were visualized by Western blotting using RSK2 and HA-specific antibodies as indicated.

### In vitro cGAS activity assay

To measure cGAS activity, Purified His-cGAS bound in HisPur Ni-NTA resin (cat #: 88221, Thermo-Fisher Scientific) were used for an in vitro kinase assay with active RSK2. A fifth of in vitro kinase reactants were used to confirm the phosphorylation of cGAS by Western blotting. The rest 4 of fifth was then turned into an in vitro cGAS activity analysis by adding the salmon sperm DNA (100 nM of double stranded DNA with average 2 kb) and 250 nM of final concentration of ATP and GTP in the reaction buffer (20 mM Tris-HCl, pH 7.5, 20 mM MgCl2, 25 mM KCl2, 100 nM ssDNA, 1 mM DTT) of cGAMP kit. The reaction was conducted at 37 °C for 1 h. The produced cGAMP content was measured by ELISA assay using the 2′, 3′-cGAMP ELISA kit (Cayman 2′3′-cGAMP ELISA kit, 501700) followed by the manufacturer’s protocol.

### Anchorage-independent colony growth assay

JB6 Cl41 and SK-MEL-2 cells stably expressing mock or cGAS were subjected to evaluate the colony growth in soft agar assay by the stimulation of growth factors, such as EGF or bFGF. The cells (8 × 10^3^) were combined with 1 ml of 0.3% agar-complete medium supplemented with 10% FBS and overlayed on the 3 ml of bottom agar. The plates were then incubated at 37 °C in a 5% CO_2_ incubator until colonies were grown to the appropriate size (generally 14–21 days). The colonies were scored using an ECLIPSE Ti inverted microscope and the NIS-Elements AR (V. 4.0) computer software program (Nikon Instruments Korea, Gangnam, Seoul, Korea), as previously described [60].

### Cell cycle synchronization and analysis by FACS

SK-MEL-2 cells and SK-MEL-2 stably expressing pCDH-mock, pCDH-cGAS, pCDH-RSK2, pCDH-cGAS+ pCDH-RSK2 cells (4 × 10^5^) were seeded into 60-mm dishes and cultured for 24 h at 37 °C in a 5% CO_2_ incubator. To synchronize the cell at G_2_/M phase, the cells were treated with nocodazole (0.2–1.0 μM) for 24 h. The cells were trypsinized, harvested, fixed with ice-cold 70% ethanol for 2 h, and treated with RNase A (200 μg/mL; Sigma-Aldrich) for 40 min at room temperature. The cells were then stained with propidium iodide (PI; 20 μg/mL; Sigma-Aldrich) for 15 min at 4 °C and subjected to flow cytometry analysis using a BD FACSCalibur™ flow cytometer (BD, Franklin Lakes, NJ, USA). The cell population was measured using Modfit LT (ver3.3) computer program.

### Statistical Information

The data are reported as the mean ± standard error of the mean (SEM). Statistical analysis was performed using GraphPad Prism 8 and R, with details regarding sample sizes, experiment reproducibility, and statistical tests provided in the Figure legends. The Student’s *t* test was used to compare values between two different groups. *, *p* < 0.05; **, *p* < 0.01; ***, *p* < 0.001. The Student’s *t*-distribution was used for the Pearson correlation significance test. *, *p* < 0.05; **, *p* < 0.01; ***, *p* < 0. 001. The Wilcoxon test was used for non-parametric significance testing between the two groups. ns, *p* > 0.05; *, *p* < 0.05; **, *p* < 0.01; ***, *p* < 0.001; ****, *p* < 0.0001.

## Supplementary information

Supplementary Table and Figures

Whole blots for the Western blotting

## Data Availability

All datasets are available from the corresponding authors upon reasonable request.
