# Supplementary material for: RSK2-mediated cGAS phosphorylation induces cGAS chromatin-incorporation-mediated cell transformation and cancer cell colony growth
Source: Cell Death Discov. 2024 Oct 18;10:442. doi: 10.1038/s41420-024-02208-8 (PMC11492232; doi:10.1038/s41420-024-02208-8)
Supplement: Supplementary file 1 — Supplementary Table and Figures [file 41420_2024_2208_MOESM1_ESM.pdf]

[Supplementary Information]

RSK2-mediated cGAS phosphorylation induces cGAS chromatin-incorporation-mediated cell transformation and cancer cell colony growth

Weidong Chen[1], Ga-Eun Lee[1,5], Dohyun Jeung[1], Jiin Byun[1], Wu Juan[1], Xianzhe Li[1], Joo-Young Lee[1,3], Han Chang Kang[2,3], Hye Suk Lee[1,3], Kwang Dong Kim[4], Soo-Bin Nam[1,5], Cheol-Jung Lee[5], Young Jik Kwon[2,6], and Yong-Yeon Cho[1,3,*]

Contents:

1. One Supplementary Tables: Supplementary Table 1

2. Six Supplementary Figures: Supplementary Figure 1-6

Supplementary Table 1 Detailed bonds and interaction types and amino acids involved in cGAS and RSK2 interaction. The table includes types of bonds (Hydrogen, Electrostatic, Hydrophobic), specific amino acids in RSK2 and cGAS involved in these interactions, and bond distances calculated using the Discovery Studio software (ver. 2021). The crystal structures of wild-type RSK2 and cGAS were obtained from the Protein Data Bank (PDB).

ZDock Score between RSK2 and cGAS			
ZRank Score	-59.806 kcal/mol		
Interaction type	amino acid		Distance (Å)
	RSK2	cGAS	
Hydrogen bond	THR139	LEU310	3.39
	HIS60	LYS315	2.29
	LYS116	SER317	2.45
	GLU140	LEU310	3.18
	ARG110	Lys421	2.51
	LEU214	Lys425	2.97
	LYS116	ASP319	3.55
Electrostatic interaction	LYS57	Glu314	5.08
	LYS104	ASP319	4.59
	ARG110	GLU422	4.39
	ARG112	GLU216	4.98
	LYS116	GLU225	5.57
Hydrophobic interaction	LYS104	LEU310	4.07
	ARG110	LYS421	4.09
	ARG114	LEU310	4.95
	HIS60	LYS315	5.06

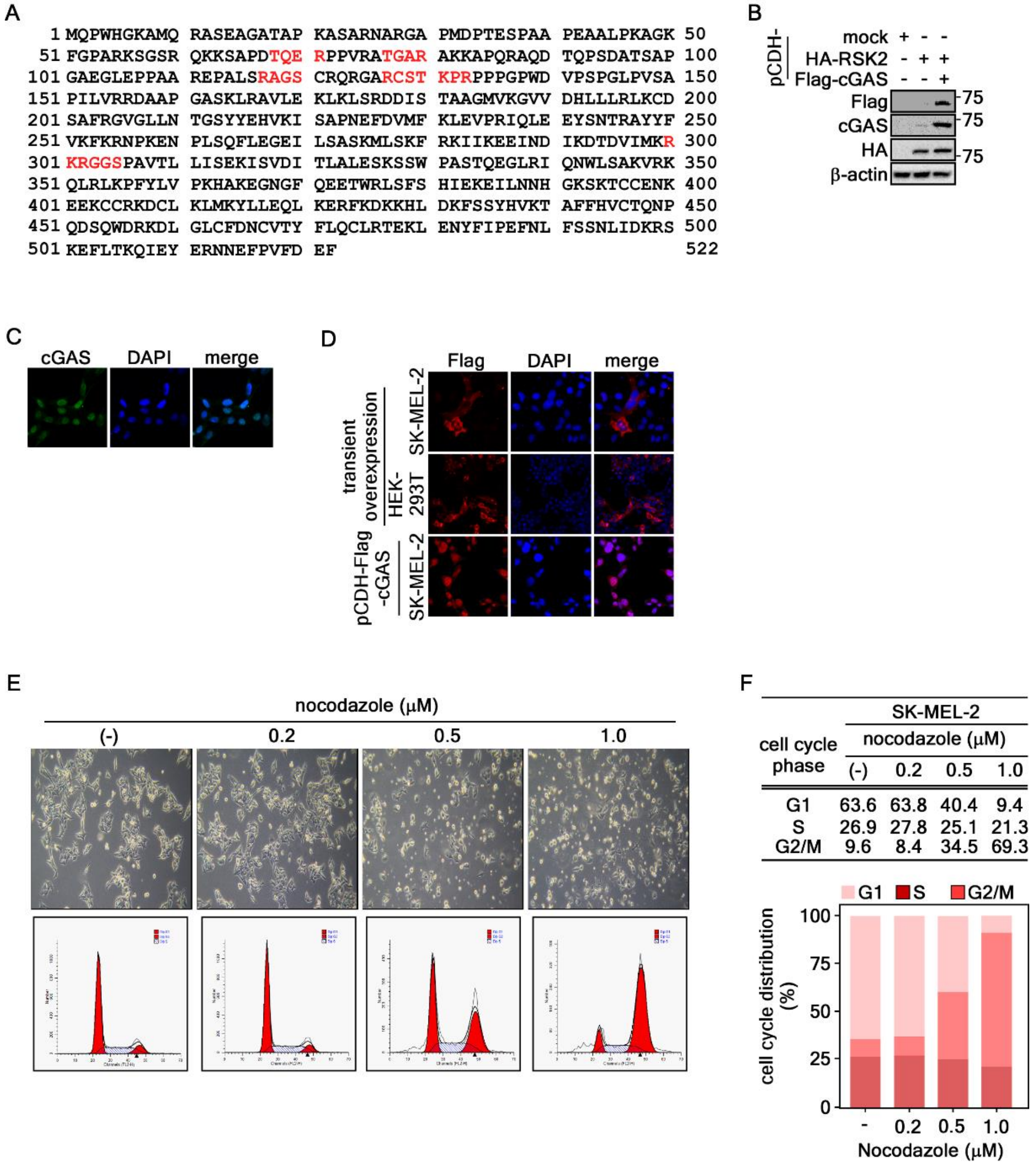

Supplementary Fig. 1 Non-canonical observation that endogenous expressing and stably overexpressing cGAS are mainly localized at the nucleus. **A** Amino acid sequence of cGAS. Red colors, putative RSK2 target phosphorylation sites. **B** Western blots confirming the stable expression of RSK2 and cGAS in SK-MEL-2 cells. The stable SK-MEL-2 cells were used to confirm the interaction by IP/Western blotting as shown in **Fig. 1E**. **C** ICF illustrating that endogenous cGAS is predominantly localized in the nucleus in SK-MEL-2 cells. Cells were stained with specific antibodies against cGAS, detected by Alexa Fluor 488 for cGAS, and DAPI for nuclei. **D** ICF illustrating that transiently expressed cGAS proteins by transfection are predominantly observed in the cytosol not only in SK-MEL-2 cells but also in HEK293T cells. These results support the **Fig. 1D** in the main text. In contrast, stably expressed Flag-cGAS in SK-MEL-2 was shown the nuclear accumulation. **E-F** Nocodazole-induced cell cycle phase analysis in SK-MEL-2 malignant melanoma cells. **E** Illustrations confirming the dose-dependent cell morphology change (*upper panels*) and G_2/M cell cycle phase accumulation by nocodazole treatment (*bottom panels*) in SK-MEL-2 cells. Cells were stained with propidium iodide (PI) and analyzed by flow cytometry. **F** Illustrations showing the cell cycle distribution by nocodazole treatment in SK-MEL-2 cells. The determined

nocodazole concentration was used to analyze the ICF of RSK2 and cGAS in **Fig. 1F** and RSK2 and cGAS chromatin incorporation in **Fig. 1G**.

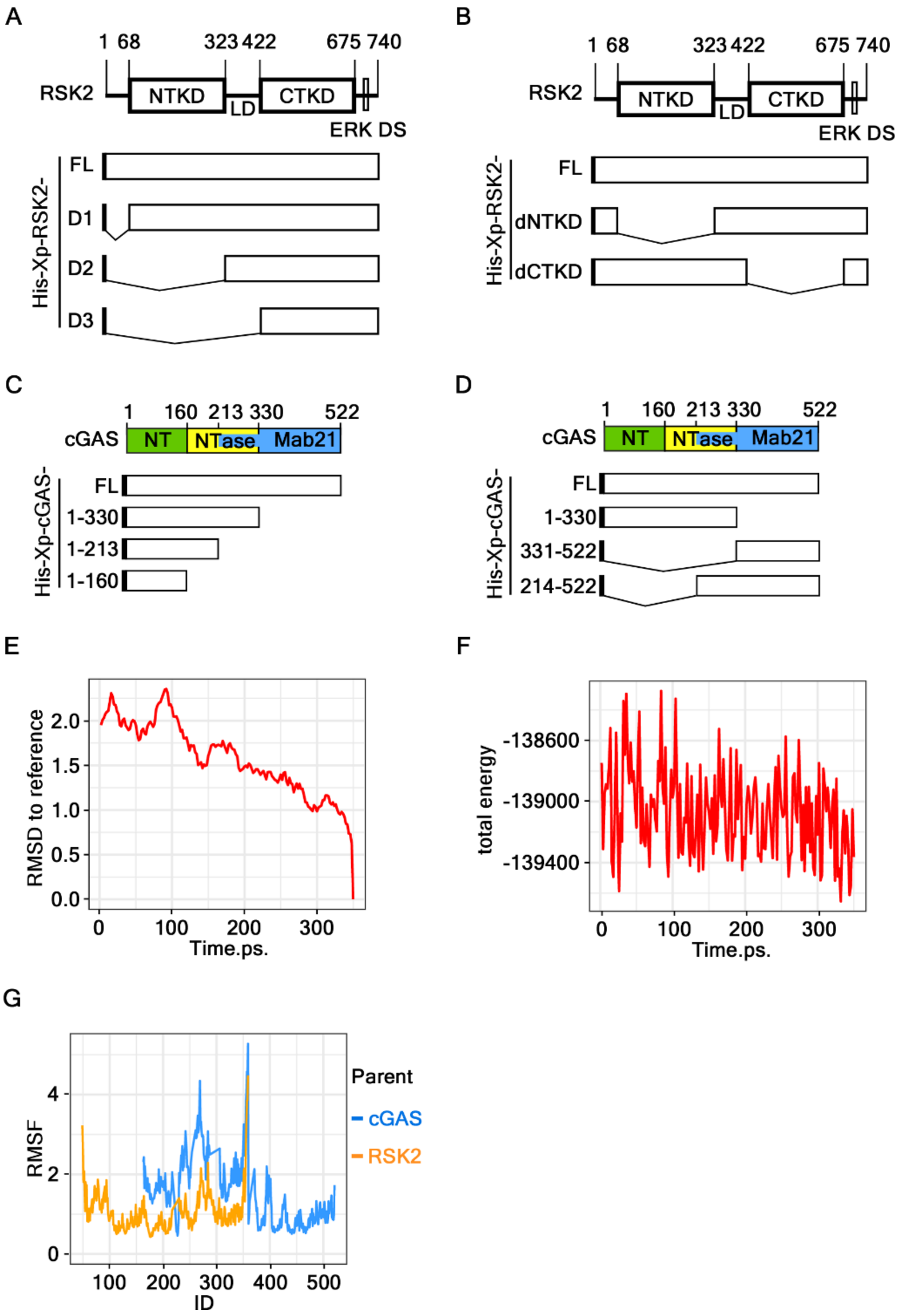

Supplementary Fig. 2 **A** Map for the serial deletion constructs of RSK2 from the N-terminal end. The serial deletion mutants are designated as His-Xp-RSK2-FL, -D1, -D2, and -D3 as indicated. NTKD, N-terminal kinase domain; LD, linker domain; CTKD, C-terminal kinase domain; ERK DS, ERK docking site. **B** Map for the kinase domain deletion mutants of RSK2. The kinase deletion mutants are designated as His-Xp-RSK2-dNTKD and d-CTKD. NTKD, N-terminal kinase domain; LD, linker domain; CTKD, C-terminal kinase domain; ERK DS, ERK docking site. **C** Map for the serial deletion mutants of cGAS from the C-terminal end. The serial deletion mutants are designated as His-Xp-cGAS-FL, -1-330, -1-213, and -1-260 as indicated. NT, N-terminal domain, NTase, nucleotidyltransferase domain; Mab21, male abnormal gene family 21. **D** Map for the specific domain deletion mutants of cGAS. The deletion mutants are designated as His-Xp-cGAS-FL, -1-330, -331-522, and -214-522 as indicated. NT, N-terminal domain,

NTase, nucleotidyltransferase domain; Mab21, male abnormal gene family 21. **E** Graph illustrating the dynamic stability of the protein-protein interface, with RMSD values measured in angstroms (Å). The root means square deviation (RMSD) of the RSK2-cGAS complex is plotted as a function of simulation time (350 ps). **F** Graph illustrating the binding process between RSK2 and cGAS over a time span of 350 ps with the total energy changes. The energy fluctuations, measured in kJ/mol, provide insights into the strength and stability of the binding interaction. **G** Graph illustrating a quantitative measure of residue flexibility during simulation of the protein interface, with RMSF values, measured in angstroms (Å). The root means square fluctuation (RMSF) individual residues within the RSK2-cGAS complex.

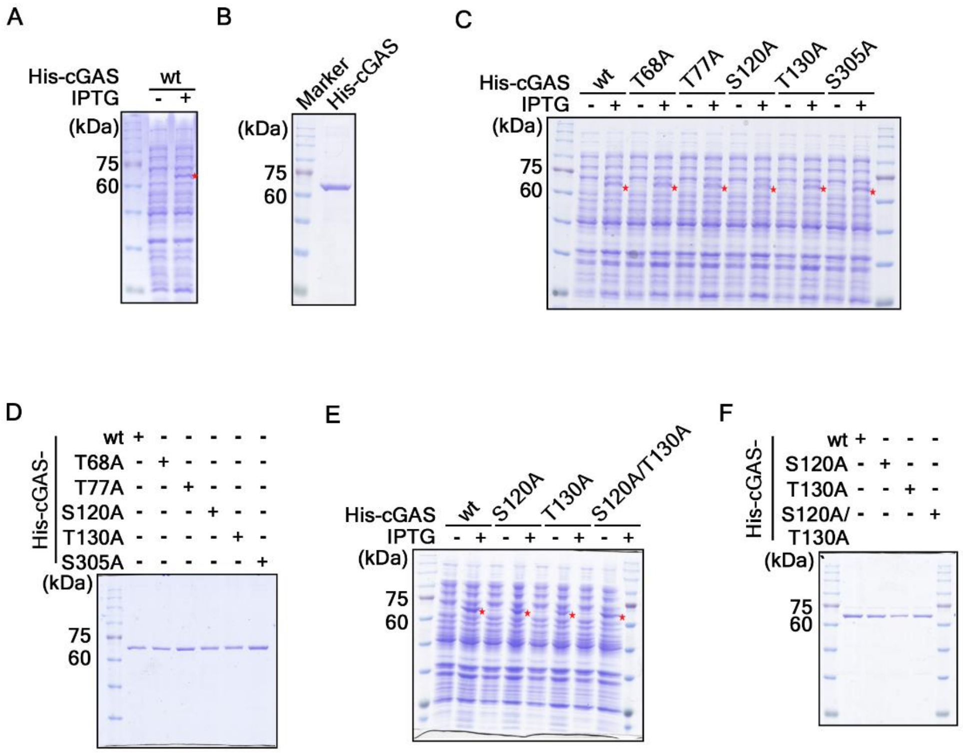

Supplementary Fig. 3 cGAS protein purification for RSK2-mediated *in vitro* kinase assay. **A-B** Purification of cGAS-wt. **A** Illustration showing Coomassie blue stained SDS-PAGE gel containing IPTG-induced wild-type cGAS protein. Star, IPTG-induced cGAS-wt. **B** Illustration indicating the purified His-cGAS protein by Ni-NTA affinity chromatography using the cell lysates in (A**)**. **C-D** Purification of His-cGAS-wt and -mt proteins. **C** Illustration showing Coomassie blue stained SDS-PAGE gel containing IPTG-induced cGAS-wt and -mt proteins as indicated. **D** Illustration indicating the purified His-cGAS-wt and -mt proteins by Ni-NTA affinity chromatography using the cell lysates in (C). **E-F** Purification of His-cGAS-wt and -mt at RSK2 target amino acids. **E** Illustration showing Coomassie blue stained SDS-PAGE gel containing IPTG-induced cGAS-wt and -mt of RSK2 target amino acids as indicated. **F** Illustration indicating the purified His-cGAS-wt and -mt proteins by Ni-NTA affinity chromatography using the cell lysates in (E).

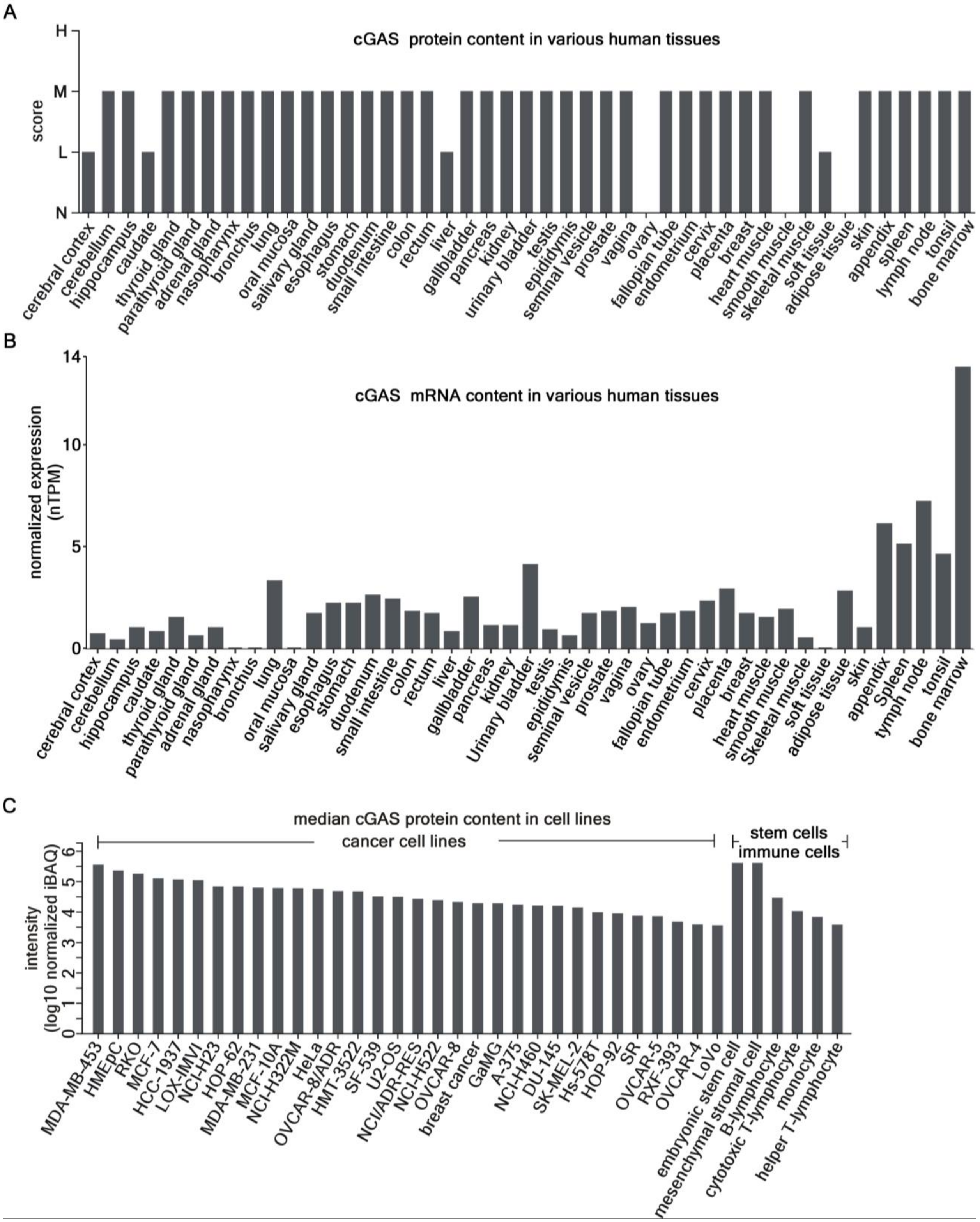

Supplementary Fig. 4 Bioinformatic analysis of cGAS protein and mRNA in various human tissues and cell lines. **A** Graphs illustrating the cGAS protein content in various human tissues. **B** Graphs illustrating the cGAS mRNA content in various human tissues. **A-B** The data set is originated from human protein atlas

(https://www.proteinatlas.org/ENSG00000164430-CGAS). **C** Graphs illustrating the cGAS protein content in various cancer and normal cell lines. The data set is originated from ProteomicsDB (https://www.proteomicsdb.org).

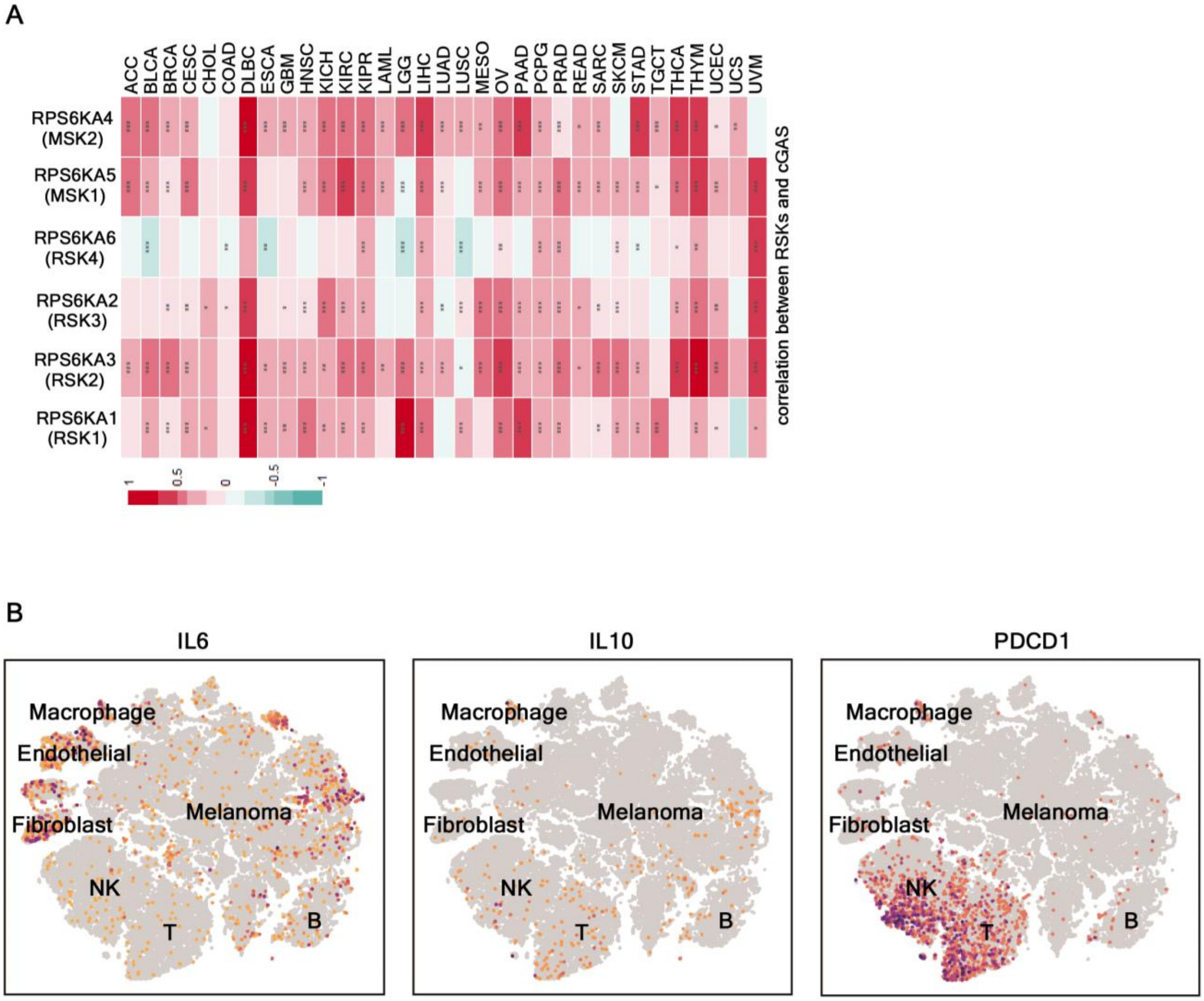

Supplementary Fig. 5 Bioinformatic analysis supporting RSK2 and cGAS association in human melanoma. **A** Analysis of mRNA expression of p90 ribosomal S6 kinase family members (RSK1-4 and MSK1-2) and cGAS using the TCGA dataset. The correlation between cGAS and each of RSK family member is denoted in color scales. The correlation with score R=0.547 is plotted in Fig. 1C as denoted in Pearson correlation coefficient. The Student's *t*-distribution was used for Pearson correlation significance test. *, $p<0.05$; **, $p<0.01$; ***, $p<0.001$. **B** Single cell RNA-seq analysis of seven datasets, including 19,200 genes from 48,638 cells, of SKCM. Immune-related ligands, including IL6, IL10, and PDCD1, are plotted. The RSK2 and cGAS are plotted in **Fig. 5D**. Statistical analysis indicated that RSK2 association in melanoma cells in the tissues was about 36.4%. Additionally, in RSK2 and cGAS association analysis, we found that cGAS and RSK2 association was observed in 6.23% among 36.4% RSK2-associated melanoma cells.

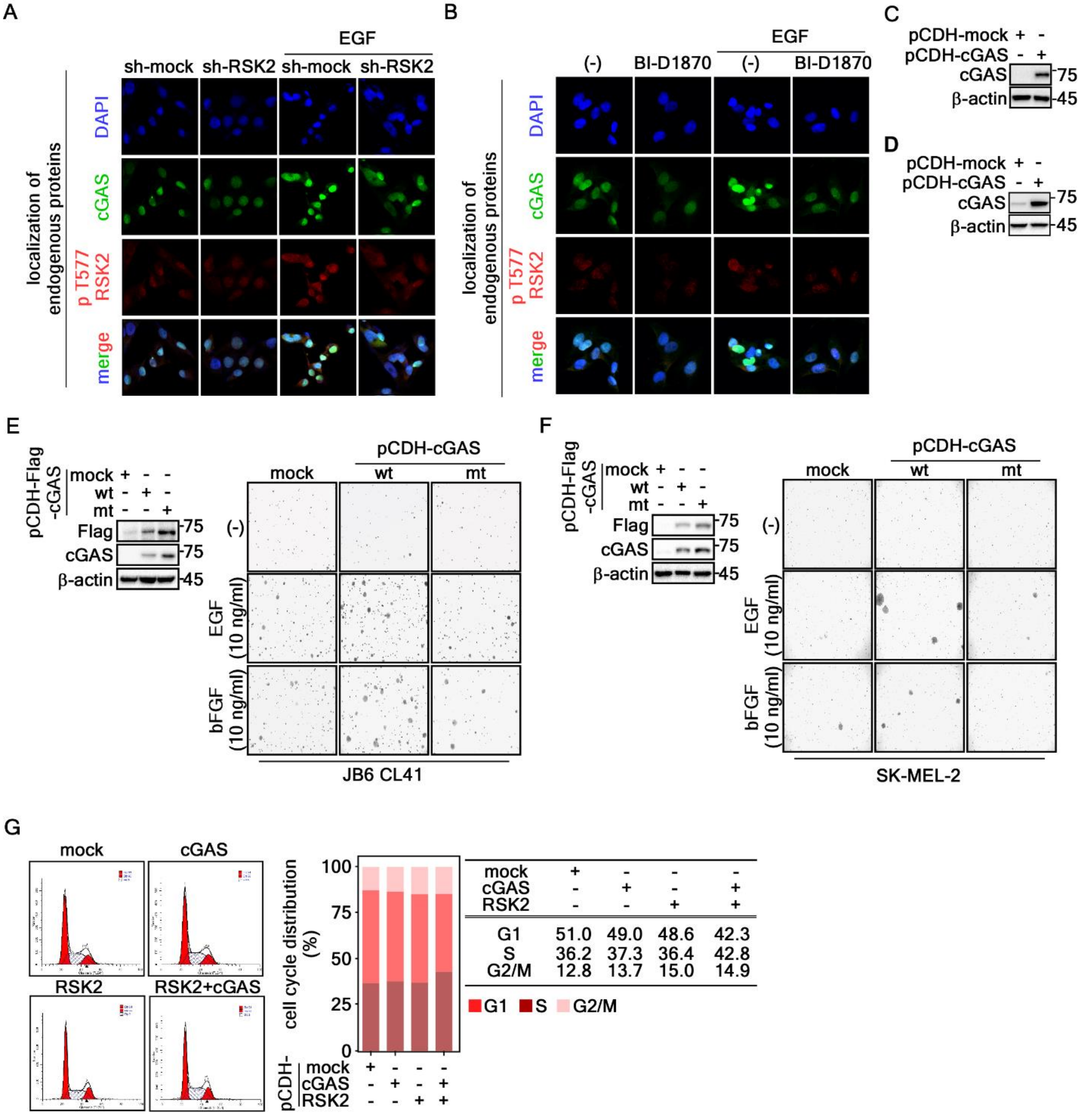

Supplementary Fig. 6 RSK2-mediated cGAS role in growth factor-induced cell transformation and colony growth of cancer cells. **A** ICF analysis indicating that the effect of RSK2 knockdown on cGAS nuclear accumulation with or without EGF stimulation in SK-MEL-2 cells. **B** ICF analysis indicating that the effect of RSK2 activity inhibition on cGAS nuclear accumulation with EGF and either BI-D1870 treatment or not in SK-MEL-2 cells. **C-D** Establishment of stable cells overexpressing cGAS-wt. **C** Western blots confirming the establishment of JB6 Cl41 cells stably expressing cGAS-wt. **D** Western blots confirming the establishment of SK-MEL-2 malignant melanoma cells stably expressing cGAS-wt. **E-F** Confirmation of the rescued cGAS-wt or -mt role in EGF- or bFGF-induced cell transformation in JB6 Cl41 cells (E) and colony growth of SK-MEL-2 cells (F). *Right panels in* (E) *and* (F) Westen blots illustrating the expression of recued cGAS-wt and -mt in JB6 Cl41 cell (E) and SK-MEL-2 cells (F). *Left panels in* (E) *and* (F) Photographs showing colony formation of the JB6 Cl41 cells induced by EGF or bFGF (E) and colony growth of the SK-MEL-2 cells induced by EGF or bFGF (F). The statistically analyzed results are provided

in Fig. 6K and L. **G** Illustrations showing the effectiveness of cell cycle distribution by stable expression of cGAS, RSK2 or cGAS/RSK2 in SK-MEL-2 cells. *Left panels*, diagrams of flow cytometry analyzed by Modifit; *Graphs* and *table*, cell population obtained by flow cytometry.
