# Supplementary material for: RSK2-mediated cGAS phosphorylation induces cGAS chromatin-incorporation-mediated cell transformation and cancer cell colony growth
Source: Cell Death Discov. 2024 Oct 18;10:442. doi: 10.1038/s41420-024-02208-8 (PMC11492232; doi:10.1038/s41420-024-02208-8)

Whole blots for the Fig. 1 by WC et. al.

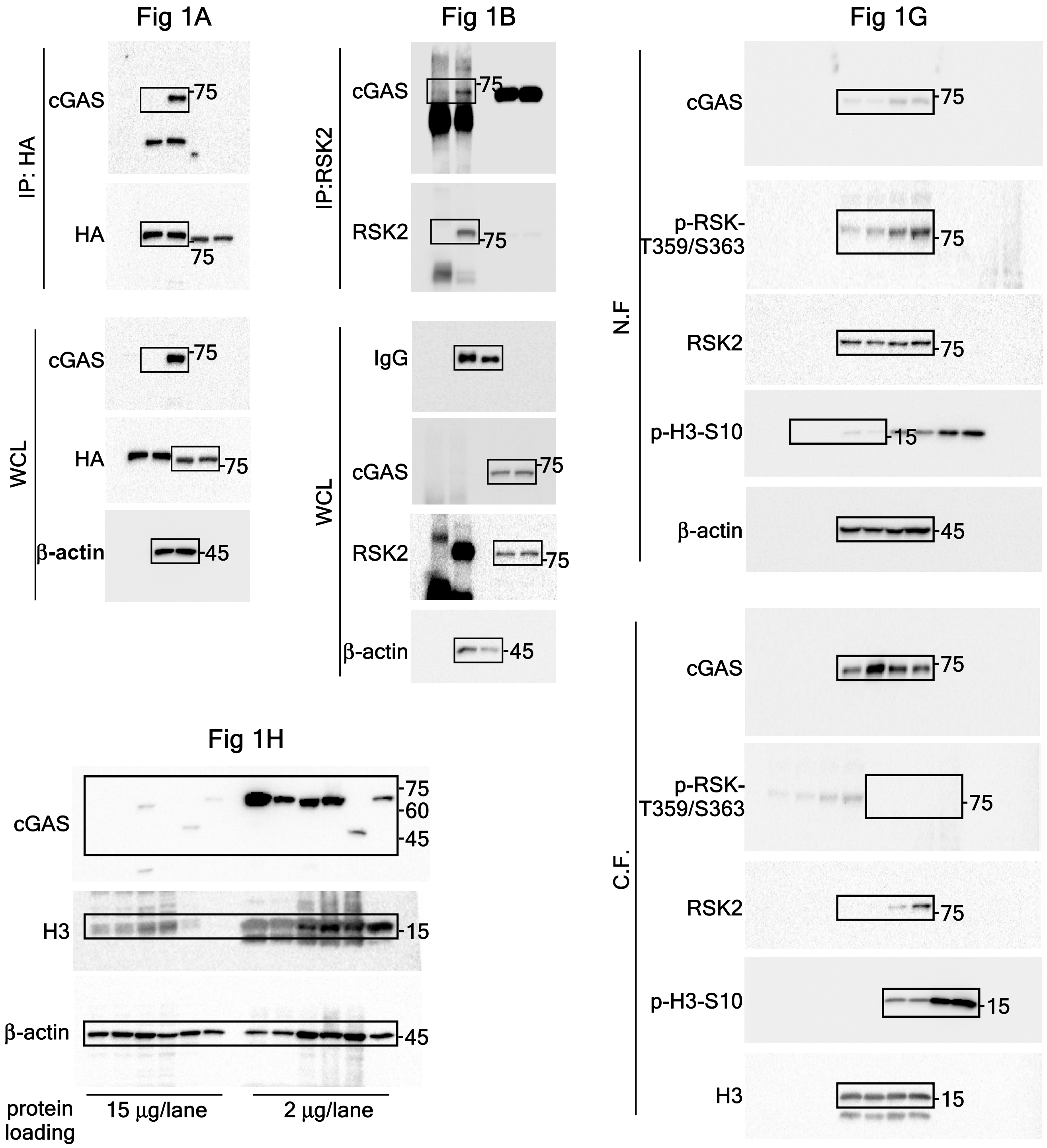

Whole blots for the Fig. 2 by WC et. al.

Fig 2A

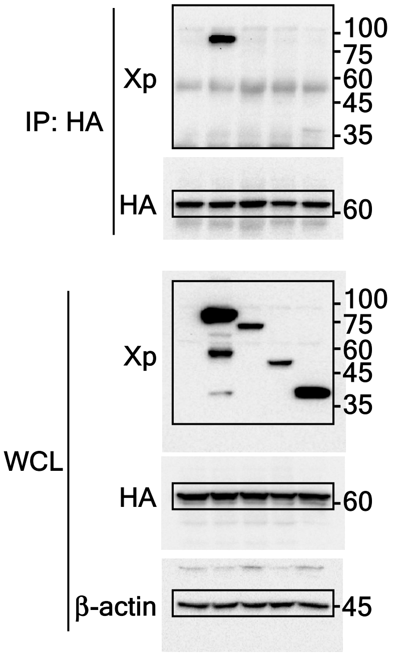

Fig 2B

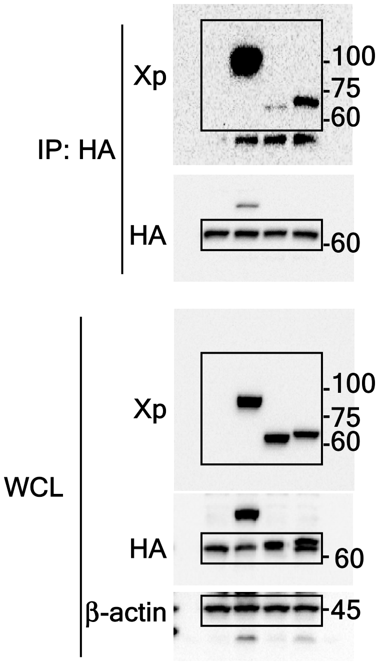

Fig 2C

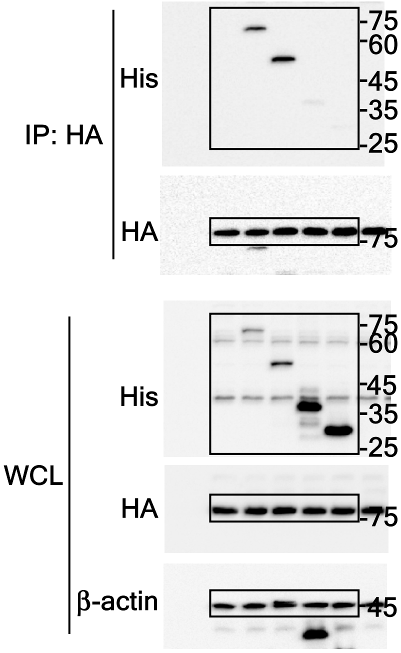

Fig 2D

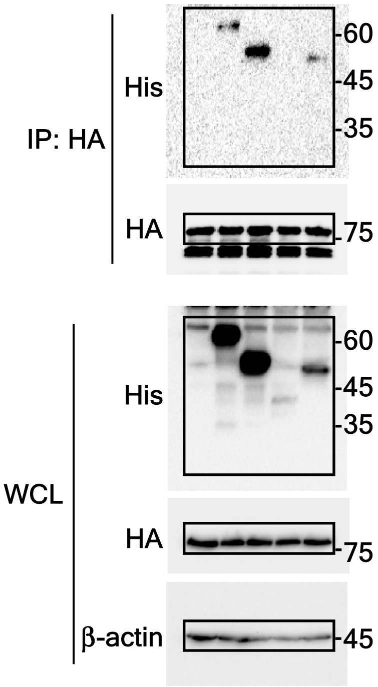

Whole blots for the Fig. 3 by WC et. al.

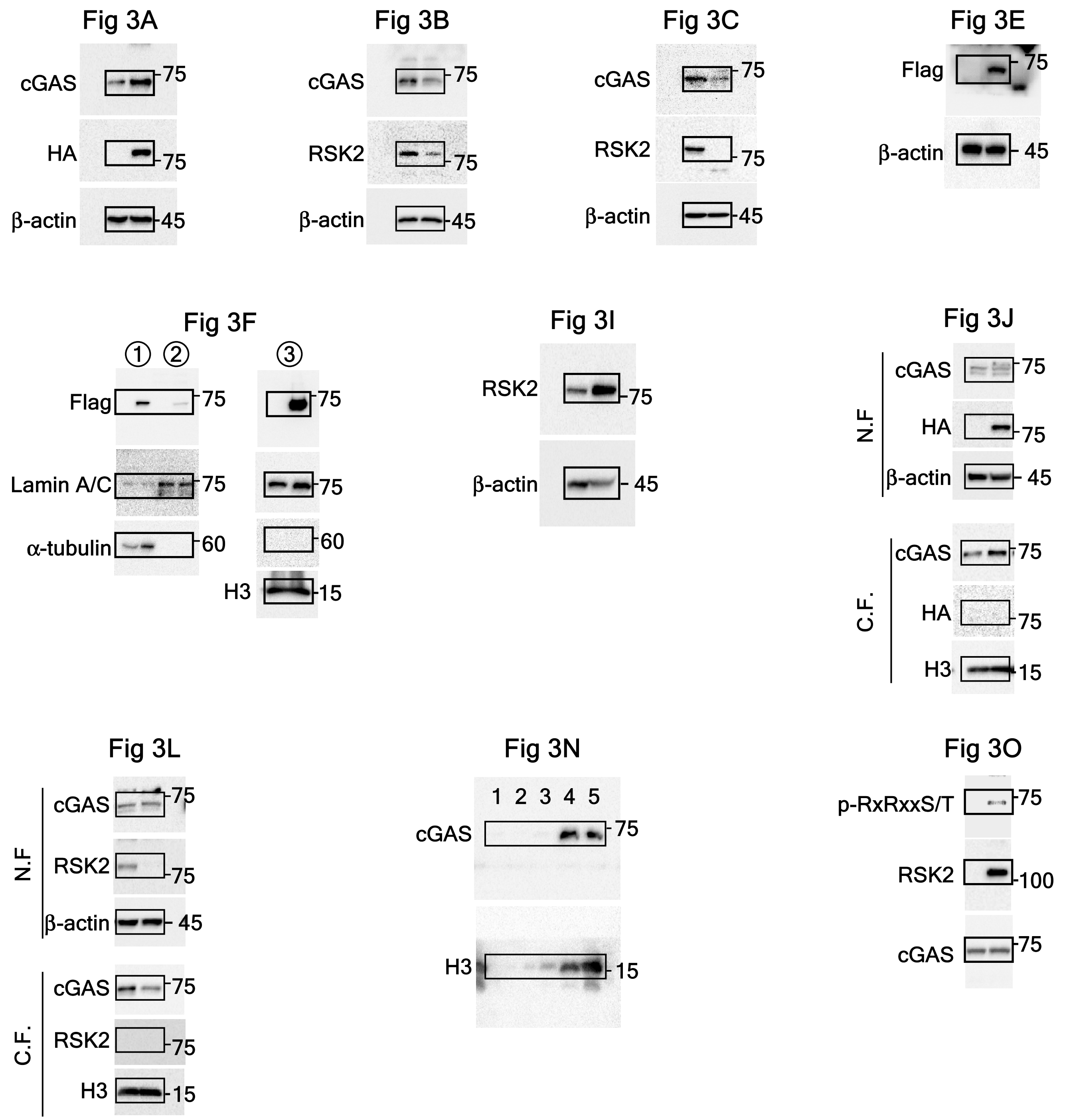

Whole blots for the Fig. 4 by WC et. al.

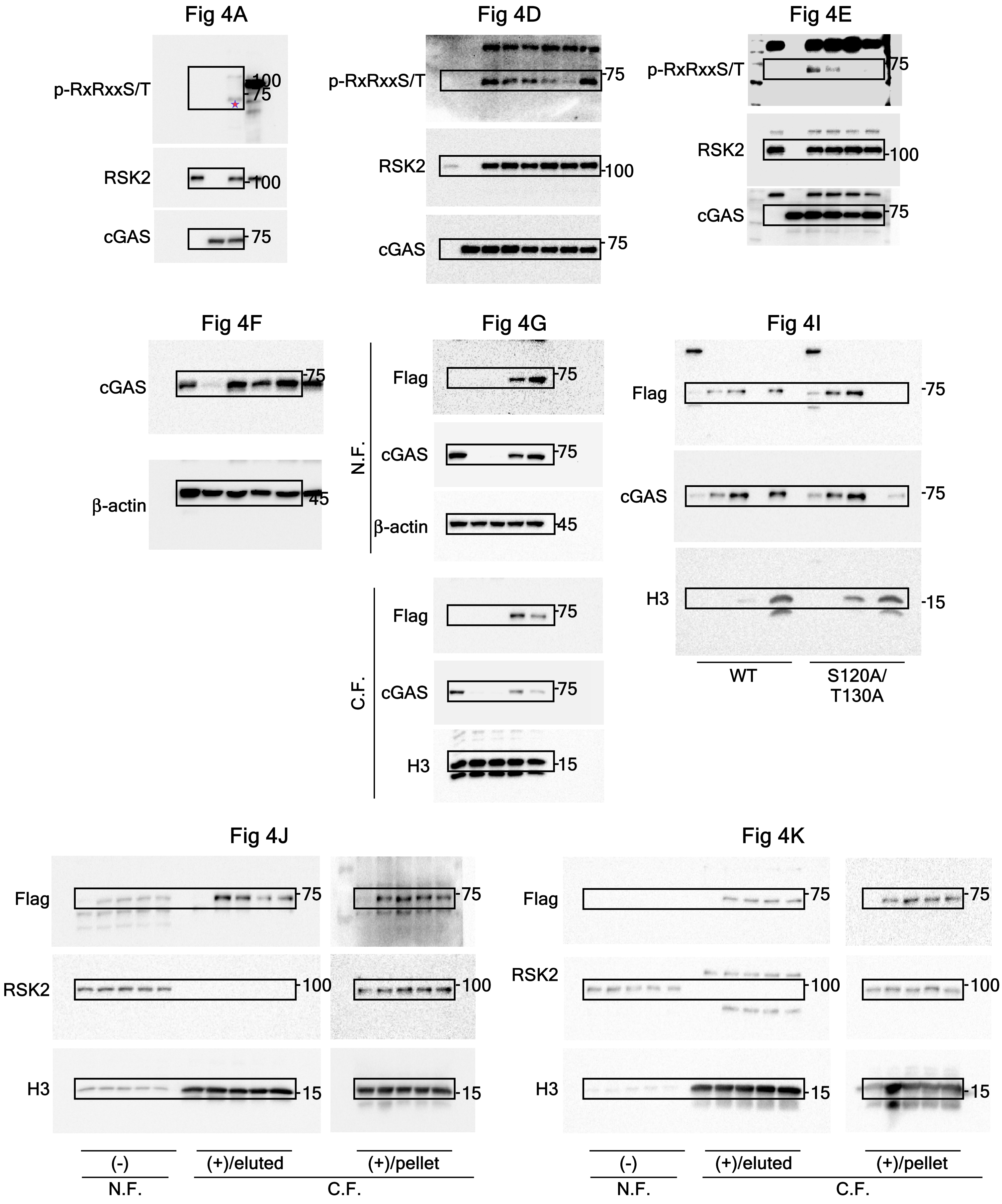

Whole blots for the Fig. 6 by WC et. al.

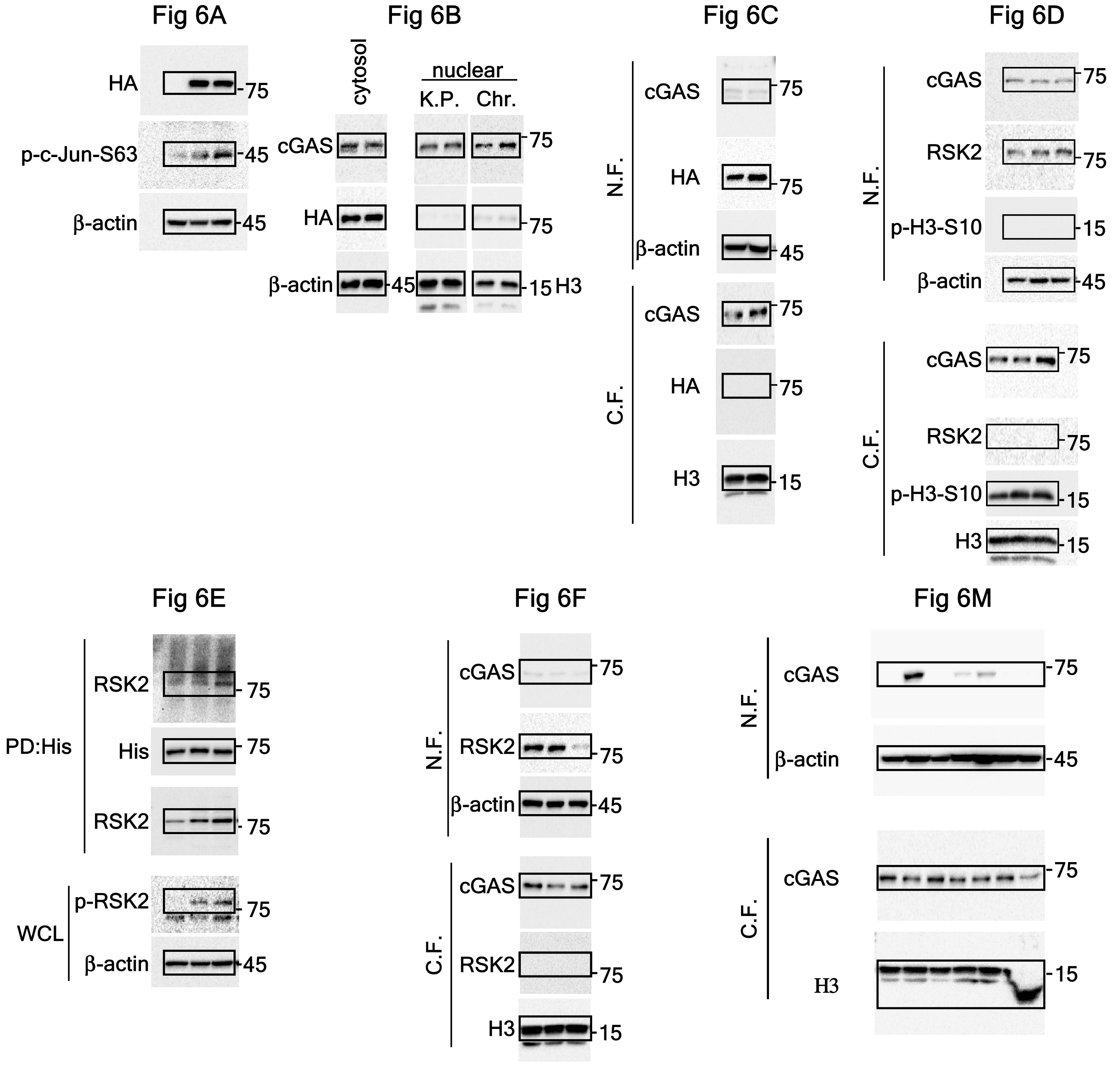

Whole blots for the Supplementary Fig. 1 by WC et. al.

Suppl Fig. 1B

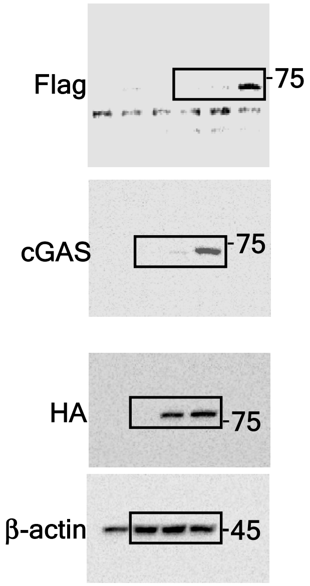

Whole blots for the Supplementary Fig. 3 by WC et. al.

Suppl Fig. 3A

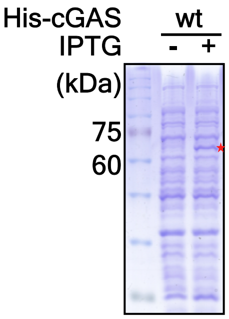

Suppl Fig. 3B

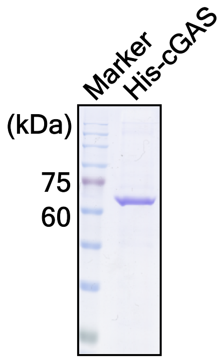

Suppl Fig. 3C

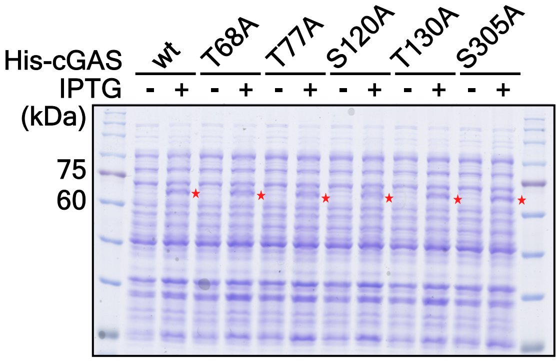

Suppl Fig. 3D

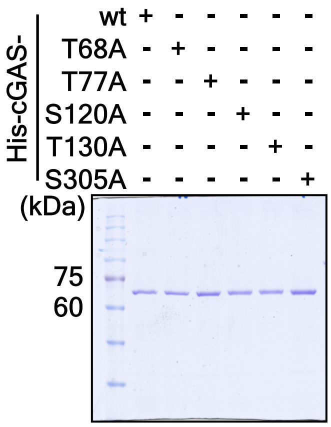

Suppl Fig. 3E

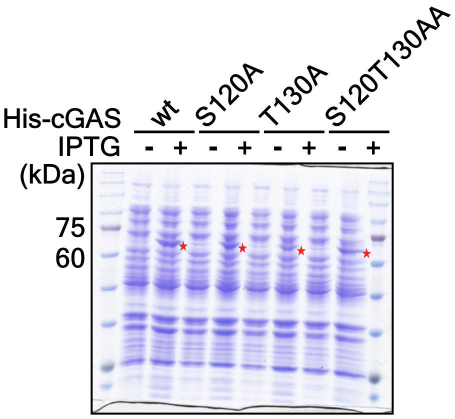

Suppl Fig. 3F

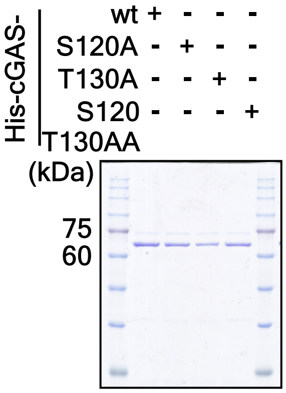

Whole blots for the Supplementary Fig. 6 by WC et. al.

Suppl Fig. 6C

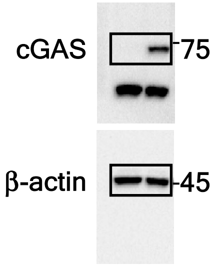

Suppl Fig. 6D

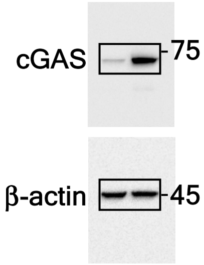

Suppl Fig.6E

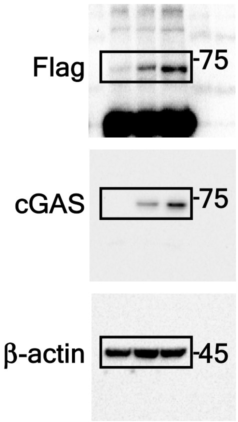

Suppl Fig. 6F

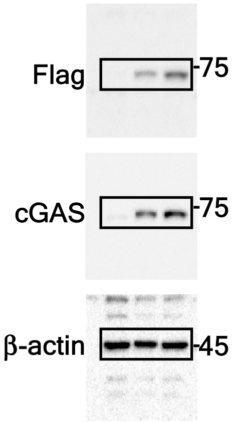

Supplement: Supplementary file 2 — Whole blots for the Western blotting [file 41420_2024_2208_MOESM2_ESM.pdf]
